# Assessing Impacts of Atmospheric Conditions on Efficiency and Siting of Large-Scale Direct Air Capture Facilities

**DOI:** 10.1021/jacsau.4c00082

**Published:** 2024-05-01

**Authors:** Xuqing Cai, Mark A. Coletti, David S. Sholl, Melissa R. Allen-Dumas

**Affiliations:** † School of Chemical & Biomolecular Engineering, Georgia Institute of Technology, Atlanta, Georgia 30332, United States; ‡ Oak Ridge National Laboratory, 1 Bethel Valley Road. Oak Ridge, Tennessee 37831, United States

**Keywords:** direct air capture, carbon dioxide removal, atmospheric conditions, facility siting

## Abstract

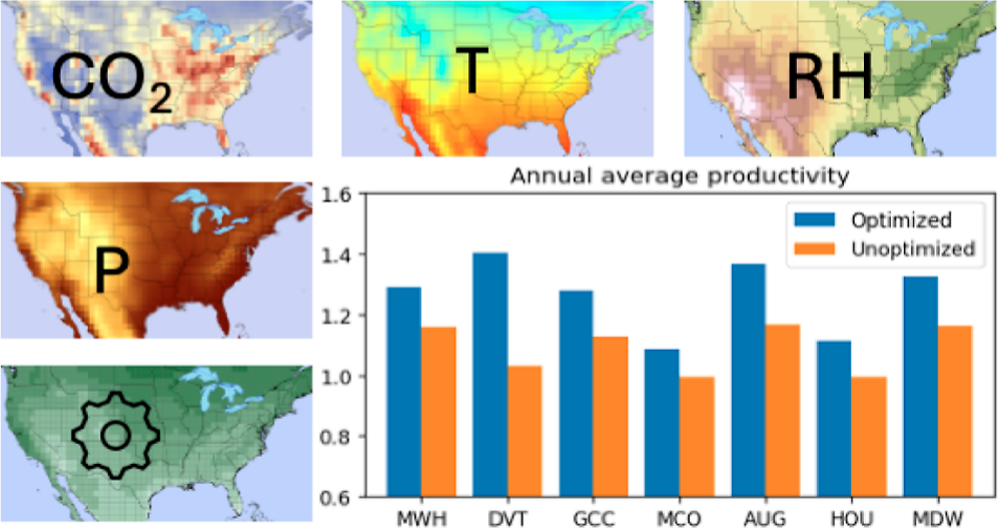

The cost and efficiency of direct air capture (DAC) of carbon dioxide (CO_2_) will be decisive in determining whether this technology can play a large role in decarbonization. To probe the role of meteorological conditions on DAC we examine, at 1 × 1° resolution for the continental United States (U.S.), the impacts of temperature, humidity, atmospheric pressure, and CO_2_ concentration for a representative amine-based adsorption process. Spatial and temporal variations in atmospheric pressure and CO_2_ concentration lead to strong variations in the CO_2_ available in ambient air across the U.S. The specific DAC process that we examine is described by a process model that accounts for both temperature and humidity. A process that assumes the same operating choices at all locations in the continental U.S. shows strong variations in performance, with the most influential variables being the H_2_O gas phase volume fraction and temperature, both of which are negatively correlated with DAC productivity for the specific process that we consider. The process also shows a moderate positive correlation of ambient CO_2_ with productivity and recovery. We show that optimizing the DAC process at seven representative locations to reflect temporal and spatial variations in ambient conditions significantly improves the process performance and, more importantly, would lead to different choices in the sites for the best performance than models based on a single set of process conditions. Our work provides a framework for assessing spatial variations in DAC performance that could be applied to any DAC process and indicates that these variations will have important implications in optimizing and siting DAC facilities.

## Introduction

Rapid and transformational technological interventions into carbon cycle management are required to limit global warming during the 21st century.^1^ To keep global average temperatures less than 2 °C higher than that of the preindustrial age, annual removal of atmospheric carbon on gigatonne scales will be necessary.^2^ Carbon capture techniques are one strategy toward achieving this goal.^3^ While the efficient capture of CO_2_ from industrial processes may best be performed at emission sites, placement of the capture infrastructure at every emission site is likely impractical and cost prohibitive. Direct air capture (DAC) of CO_2_ is one of a suite of carbon dioxide removal approaches^4^ that is being considered for large-scale deployment across the globe with the goal of achieving carbon capture on the order of gigatonnes annually.^5,6^

Analogous to the production of chemical commodities, even incremental improvements in the cost or efficiency of DAC processes will reap large dividends if DAC is deployed at climate-relevant scales. Although laboratory testing of DAC processes is typically performed with fixed input conditions, a number of studies have indicated that meteorological characteristics of ambient air can significantly affect DAC performance. Kulkarni and Sholl^7^ tested two DAC process models using hourly temperature data at six climatically different U.S. locations. Haghpanah et al.^8^ evaluated pressure swing adsorption cycles for postcombustion CO_2_ capture at different ambient pressures. An et al.^9^ showed that CO_2_ capture in a solvent-based DAC process was more sensitive to temperature than to humidity and that capture rates could be expected to vary between 10 and 90% across large temperature and humidity ranges. Kolle et al.^10^ studied the effects of water vapor on the performance of CO_2_ adsorption over four different types of adsorbent materials. Wiegner et al.^11^ investigated DAC under varying ambient temperature and humidity conditions using process optimization for an assigned set of temperature–humidity combinations.

In addition to the well-known local variations in temperature and humidity, the CO_2_ available in ambient air also varies spatially and temporally. General circulation models have shown that the spatial distribution of global CO_2_ concentration is nonuniform, and measurements show that some locations record larger annual amplitudes of the CO_2_ cycle than others.^12,13^ These inhomogeneities and variations in atmospheric pressure among locations on continental scales mean that the amount of CO_2_ available for a fixed volume of ambient air is far from uniform among geographic locations. Previous analyses of DAC process efficiency have not considered these effects in a systematic way.^5^

**Figure 1:**
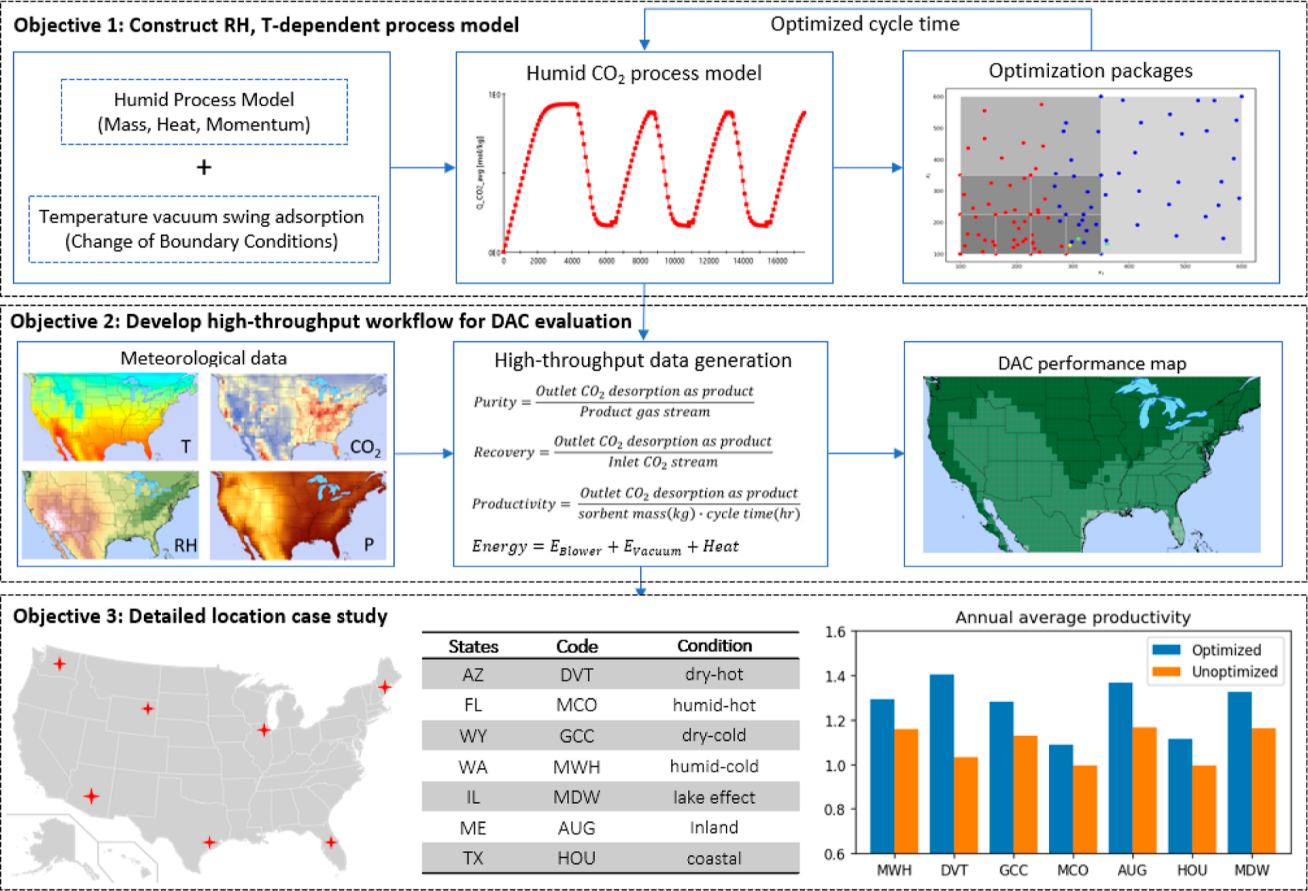
Workflow for analysis of impacts of meteorology on performance of an adsorption-based DAC process in terms of productivity and recovery.

Below, we collate data that account explicitly for meteorological conditions, including the local CO_2_ concentration, throughout the contiguous United States (CONUS) and demonstrate the implications of these conditions for a specific adsorption-based DAC process. Specifically, we evaluate for each 1 × 1° latitude/longitude grid cell in CONUS, the combination of the effects of monthly average CO_2_ concentration, specific humidity, temperature, and atmospheric pressure on working capacity, productivity, recovery, efficiency, and cost of operating a representative amine-based DAC adsorption process using a process model described in ref (14). Figure 1 illustrates the overall workflow for the remainder of this paper:

1. Defining a process model for a specific DAC process that incorporates effects of relative humidity and temperature.
2. Using a high-throughput workflow for the evaluation of the DAC model performance for conditions throughout CONUS.
3. Optimizing the performance of the specific DAC process, we considered in seven representative locations using detailed meteorological information.

## Results and Discussion

**Figure 2:**
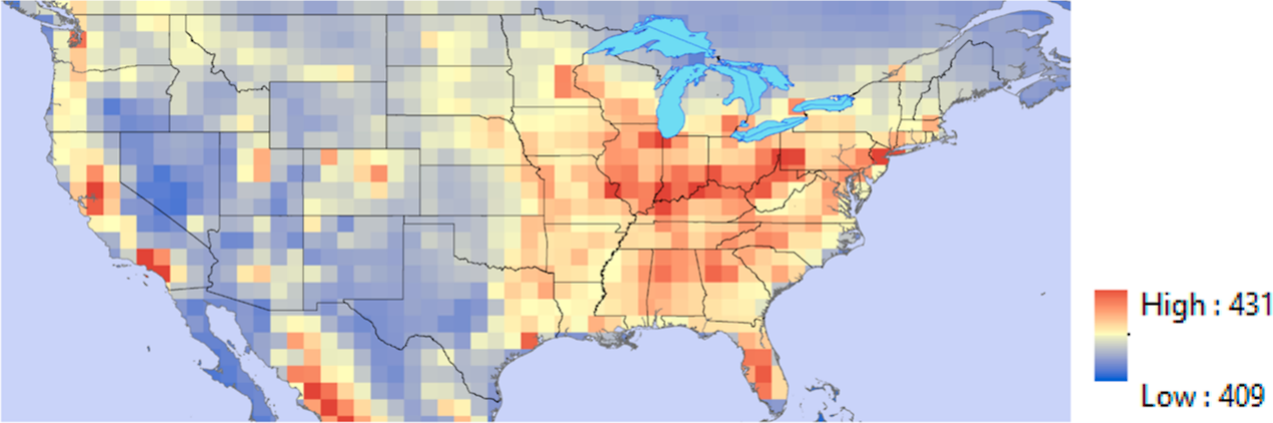
Average annual CO_2_ concentration (ppm) of the lowest atmospheric layer in each 1 × 1° grid cell for the contiguous US for calendar year 2018 using data from CarbonTracker.^13^

Meteorological data at 1 × 1° latitude/longitude grid spacing was obtained from the National Oceanic and Atmospheric Administration CarbonTracker measurement and modeling system.^13^ CarbonTracker is an inverse model of atmospheric CO_2_. That is CarbonTracker models gridded atmospheric CO_2_ by adjusting inputs and removals of CO_2_ at the surface of the Earth until they show the best agreement with observations. Data used in the experiments presented here are monthly and annual averages of values in the lowest atmospheric layer of each 1 × 1° grid cell for the year 2018. Figure 2 shows the 2018 average CO_2_ (range of annual average values is 409–431 μmol/mol) for each grid cell. In addition to CO_2_ data, specific humidity (0.002–0.016 kg/kg), temperature (250–299 K), and atmospheric pressure (71,109–102,111 Pa) were obtained for each month of 2018 from CarbonTracker and used as an input to the five-parameter DAC process model described below. Specific humidity values from CarbonTracker were converted to the water volume fraction as defined in Hartfield et al.^15^

To exemplify the impacts of meteorological conditions on DAC, we performed extensive calculations for a representative DAC process for which a model for effects of temperature, CO_2_ concentration, and humidity are available. Specifically, we considered the amine-based adsorption process analyzed previously by Elfving et al.^14^ This specific process is similar to a range of other adsorption-based DAC processes that have been tested at varying scales.^16−23^ We emphasize that this specific process was chosen because it is one of the few examples where a process model accounting for the impacts of humidity is available and not because we anticipate that it is superior to other DAC processes under development. We hope that the meteorological data we have provided in this paper and the analysis of an adsorption-based DAC process motivate others to collect the information required to allow detailed improved process modeling of many of the other DAC processes that are under active development.

To reduce the calculation time for assessing the relative significance of each of the meteorological components in the calculation of CO_2_ capture process performance in the meteorology of each 1 × 1° grid cell over the contiguous United States, we applied the humid process model for 25,344 representative sample points within the pressure-R.H.-CO_2_ concentration–temperature parameter space at a fixed set of operating parameters (adsorption time, desorption time etc.). These operating parameters were selected by optimizing the process performance under “standard” conditions, as described in the Supporting Information, so they represent choices that might be made in typical laboratory development of a process of this kind. The range and number of points included in the sampling space were based on the full range of observed meteorological conditions for each variable (Table S5). To evaluate CO_2_ capture process performance, we used four performance metrics: productivity , recovery , electricity requirements , and heat requirements . These four metrics have been used extensively in the carbon capture community to evaluate the process efficiency and operational cost.^24−26^

### Assessing Annual DAC Performance in the U.S

**Figure 3:**
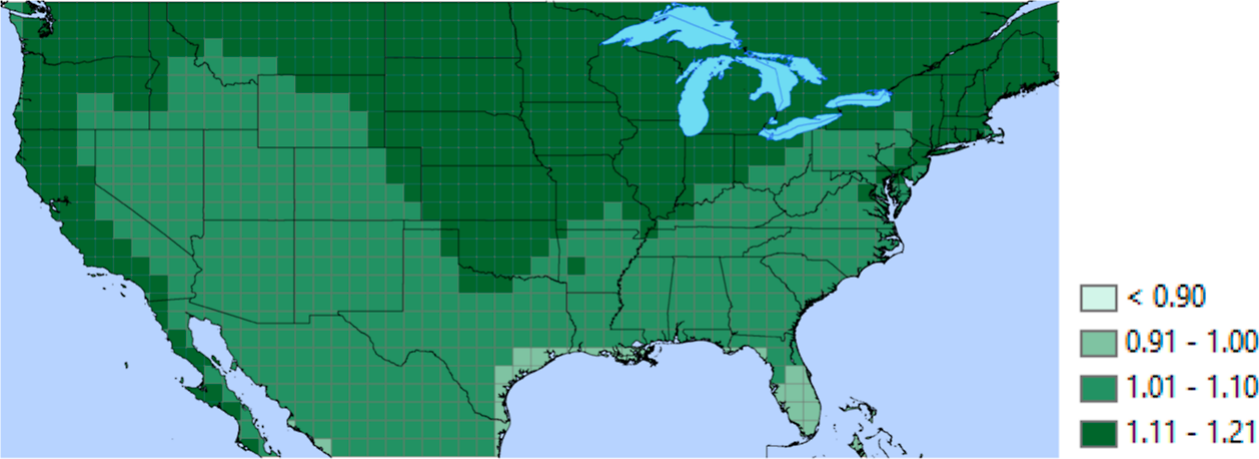
Annual average CO_2_ productivity (mol CO_2_/kg sorbent/h) over CONUS of 2018 from interpolation calculations using meteorological variables at 1 × 1° latitude/longitude grid spacing for the adsorption-based DAC process defined in the text with fixed adsorption and desorption cycle times.

Once gPROMS simulations were completed at the 25,344 input conditions described above, interpolation was used to define the performance metrics of the DAC process at other input conditions. At each grid location within the US, the DAC process performance was calculated in this way at a monthly frequency. Examples are shown in Figures S6–S10. All the data from the gPROMS uniform sampling calculations, as well as the interpolated performance data with corresponding meteorological inputs, are provided in the Supporting Information. The annually averaged DAC performance as a function of location is shown in Figure 3. It is striking that the productivity of the modeled process varies by more than 30% among locations within the US, with the lowest values in the Gulf Coast regions of Texas and Louisiana and in Florida. The productivity values seen in Figure 3 are similar to the upper range of productivities reported in a study of a similar adsorption-based DAC process by Sabatino et al.^27^ This work also included some cases with considerably lower productivity (as low as 0.25 mol CO_2_/kg sorbent/h) for variations of their process with much lower heat requirements than the process we considered. For a process that is envisioned for use at very large scales with intense attention to cost efficiency, this is a large effect, underscoring the importance of considering spatial variations among potential DAC sites.

**Figure 4:**
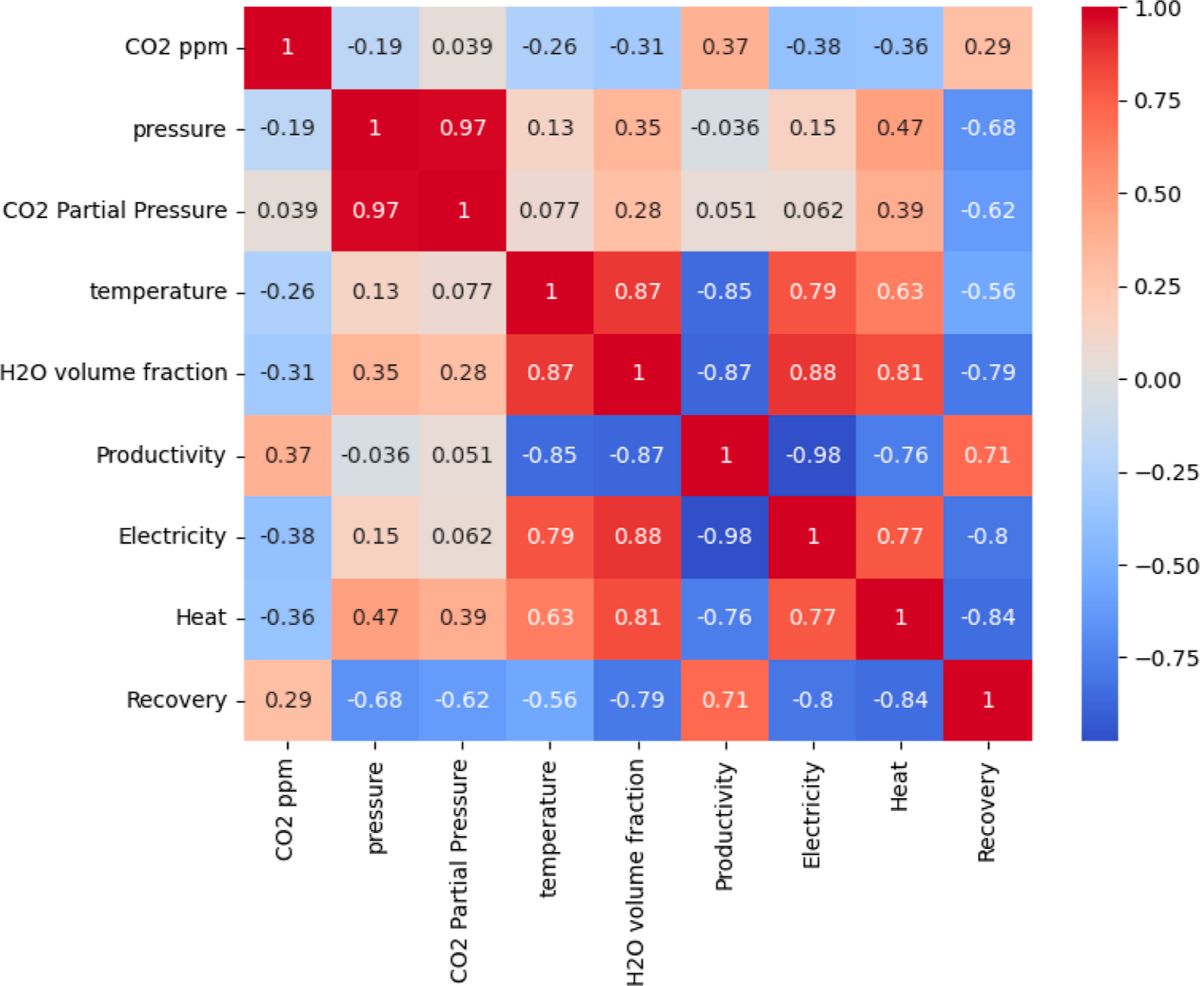
Correlation matrix of each meteorological input with various measures of DAC performance averaged across locations within the US using process simulations at constant cycle times. The four parameters at the bottom of the vertical axis and the right of the horizontal axis are the DAC performance parameters. CO_2_ partial pressure is also included to quantify the CO_2_ amount within each grid cell.

It is useful to examine what factors contribute most strongly to the variation in the DAC performance among locations. This comparison can be made using the input meteorological variable maps in Figure S3 and the correlation matrix in Figure 4. For the specific process, we modeled, the most influential variables are the H_2_O gas phase volume fraction and temperature, both of which have negative correlations with DAC productivity. Because the adsorption-based DAC process is an exothermic process and the regeneration temperature was fixed for all locations, higher ambient temperature led to a lower temperature swing and decreased the overall working capacity of the DAC process. This effect can also be seen in the monthly productivity plots in Figure S6, which show that quarter 3 (July, August, and September) has lower productivity than quarter 1 (January, February, and March).

Because the H_2_O volume fraction is a direct measurement of water content of the atmosphere, it was used to study the influence of water to the DAC process instead of relative humidity. Previous studies have found adding H_2_O during adsorption with amine-adsorbent-based materials can increase amine efficiency by reducing the number of amine groups required for CO_2_ adsorption.^10,28−31^ Higher humidity, however, can also have a negative impact on the process performance. At higher H_2_O gas phase volume fractions, longer desorption cycles are required for complete H_2_O and CO_2_ desorption. In particular, high H_2_O loadings can impede the initial desorption of CO_2_. This means that in our case study with fixed cycle times there is a reduction in net CO_2_ desorption at higher H_2_O volume fractions. In addition, high H_2_O gas phase volume fractions in ambient air are strongly correlated with the temperature, and in the process, we considered higher ambient temperatures leads to lower productivity.

Figure 2 shows that the annual average CO_2_ concentration in the US varies by approximately 5%. Figure 4 shows that there is a moderate positive correlation between the CO_2_ concentration and DAC productivity. A potentially more process-relevant quantity is the partial pressure of CO_2_ at each location, which defines the maximum amount of CO_2_ available from a fixed volume of air passing through a DAC process. This annually averaged partial pressure variation is shown in Figure S4 and has a broader range (29–43 Pa) than the CO_2_ concentration (409–431 ppm). For the specific adsorbent we considered, the CO_2_ uptake at 298 K and 64% RH is 0.76 mol/kg with CO_2_ partial pressures of 29 Pa and 0.85 mol/kg when the partial pressure is 43 Pa. Not surprisingly, the CO_2_ partial pressure is very strongly correlated with atmospheric pressure (see Figure 4), which is in turn very strongly correlated with altitude. Despite the large variations in the CO_2_ partial pressure, the correlation between this pressure and DAC productivity for the specific process we considered is small (see Figure 4). This occurs because higher altitude locations are on average cooler and drier than lower altitude locations and for the amine-adsorbent-based process, we modeled the performance advantages of cooler and drier locations to compensate for the reduced CO_2_ per volume of air at these locations. It is important to note that for other DAC processes this outcome could be very different; a process that had higher productivity under warmer more humid conditions would likely see very large performance decreases associated with the locations with low CO_2_ partial pressures, as shown in Figure S4.

The productivity of a DAC process is, of course, not the only metric that will determine whether a process is economically viable. Figure S5 shows the annually averaged recovery, electricity requirements, and heat requirements for the process we simulated throughout CONUS. The geographic variation in electricity requirements per tonne of CO_2_ are relatively small, as this is determined by the pressure drop and the amount of CO_2_ produced. There are large variations in heat requirements, however, with more humid regions requiring more process heat. Comparing grid cells in Arizona and Georgia, for example, shows that the latter locations require roughly double the heat requirements of the former to capture one tonne of CO_2_. Figure 4 shows that heat requirements and CO_2_ productivity are negatively correlated. This primarily occurs because the heat requirements increase with H_2_O volume fraction because of the need to desorb water. As already noted, for the fixed cycle times we have considered that the CO_2_ productivity in general decreases with increased H_2_O volume fractions. This observation is also seen in Figure 4 by the strong positive correlation between the heat requirements and atmospheric water content.

We investigated the electricity and heat requirements for the four representative sets of ambient conditions with constant CO_2_ concentration and pressure (*P*_amb_ = 105 kPa and *C*_CO_2_,amb_ = 400 ppm) listed in Table S6, giving the results shown in Figure S10. To simplify the presentation of energy requirements, we unified the energy associated with thermal and electrical energy using a conversion factor of 1 GJ = 277.78 kW h. In process costing, it is important to consider thermal and electrical energy separately. Figure S10 indicates the energy requirements for different aspects of the process (in kW h/t of CO_2_), so the source of energy for each aspect could be considered in future extensions on this approach. The total energy requirements for the humid-hot condition are more than 16,000 kW h/t CO_2_ compared to 3000 kW h/t CO_2_ for the dry-cold condition. The majority of the increase in heat requirement between these two conditions is associated with the desorption of water from the adsorbent.

We emphasize that the performance metrics we have discussed are associated with the specific adsorption-based DAC process we modeled, so the geographic trends we observed should not be assumed to hold for DAC processes based on very different process concepts. Nevertheless, the strong variations in performance for this specific process among geographic locations indicate that performing an analogous analysis of geographical variability should be a standard practice in assessing the potential viability of DAC processes.

### Detailed Optimization of Process Conditions

A sensible objection to the results above is that they consider a DAC process with process conditions (e.g., cycle times for TVSA, desorption temperature) that are fixed at every location and in time. This choice is not so different from many reports of DAC in the scientific literature, in which some set of input and/or process conditions are held constant, while some aspects of the underlying process (for example, the identity of the adsorbent) are varied. Nevertheless, in developing practical DAC processes, it will be important to optimize the performance of a process to local conditions. Figure 3 emphasizes how performing this optimization for different locations is important, and Figures S6–S9 emphasize how it is additionally important to perform optimization under temporally varying conditions at different locations.

**Figure 5:**
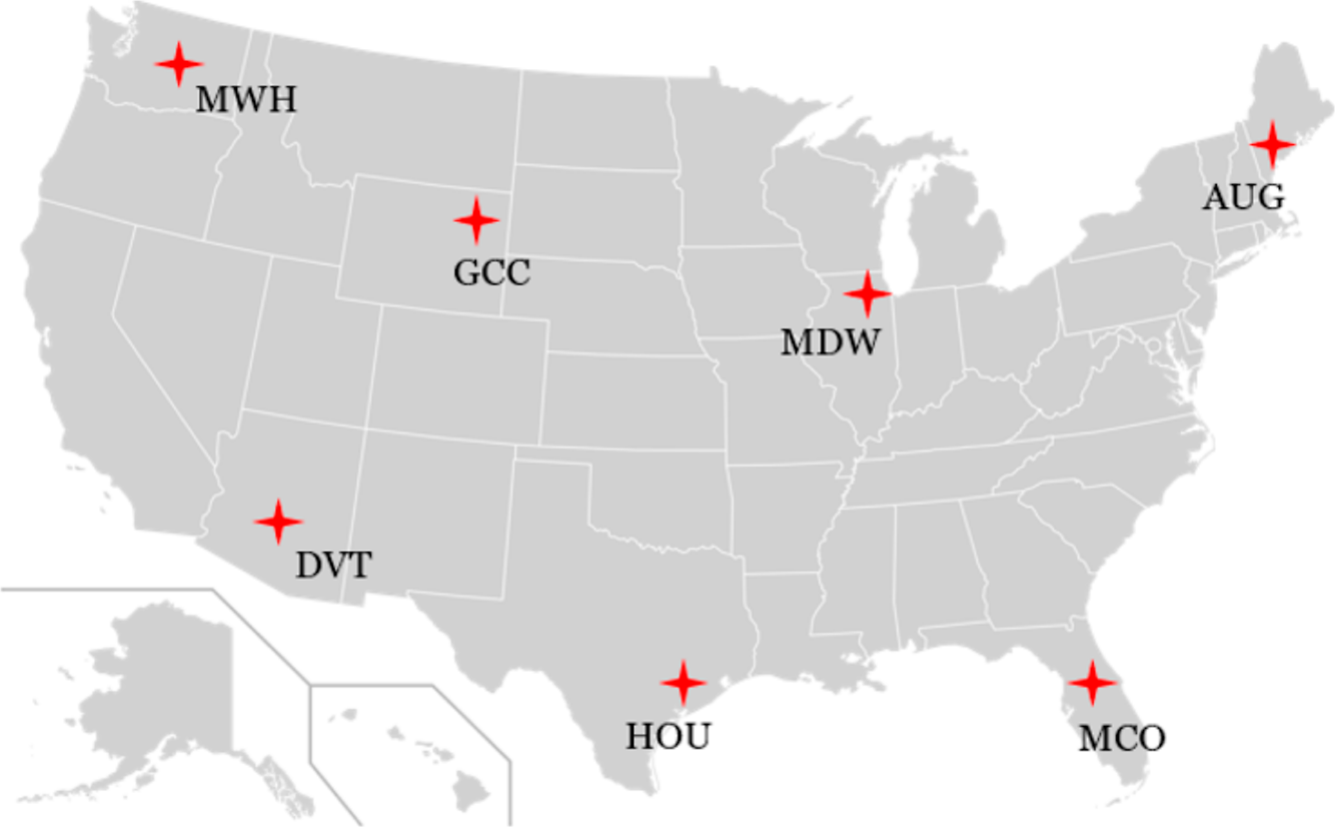
Seven representative locations in CONUS chosen for process condition optimization.

To illustrate this idea, we chose seven specific locations with different meteorological conditions from the CONUS (Figure 5 and Table S7). At each location, we optimized DAC performance for the adsorption-based process described above in two distinct formulations. In the first formulation, we maximized productivity while placing constraints on the costs associated with electricity and heat as follows
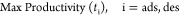

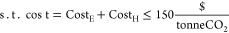

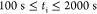


The optimization variables considered, *t*_ads_ and *t*_des_, are the adsorption and desorption time, respectively. The bounds on these times were chosen to reflect typical processes and we did not explore the sensitivity of our results to these bounds. Electricity heat costs were determined from the electricity energy requirements and heat requirements from the process model outputs assuming $0.05/kW h for electricity cost and $0.015/kW h for heat cost, independent of location, respectively. This description neglects any potential savings of heat that could be achieved by heat recovery. It would also be possible to further optimize the process by considering additional decision variables such as the vacuum pressure used for desorption.

The second formulation minimized the total energy cost defined in the same way as above to capture per tonne of CO_2_ as follows
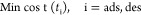

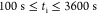


The costs here are only one contribution to the total cost of a DAC process because a full cost model must include capital expenses and other relevant factors.^11,26,27,32,33^ As such, the cost estimates reported here should be viewed as lower bounds on the process cost and not accurate estimates. The optimization for both formulations were performed the same way as determining the process conditions for high-throughput data collection: cycle times were optimized using the DDSBB package based on calculations of the quantities in the objective functions in the gPROMS process simulator.

**Figure 6:**
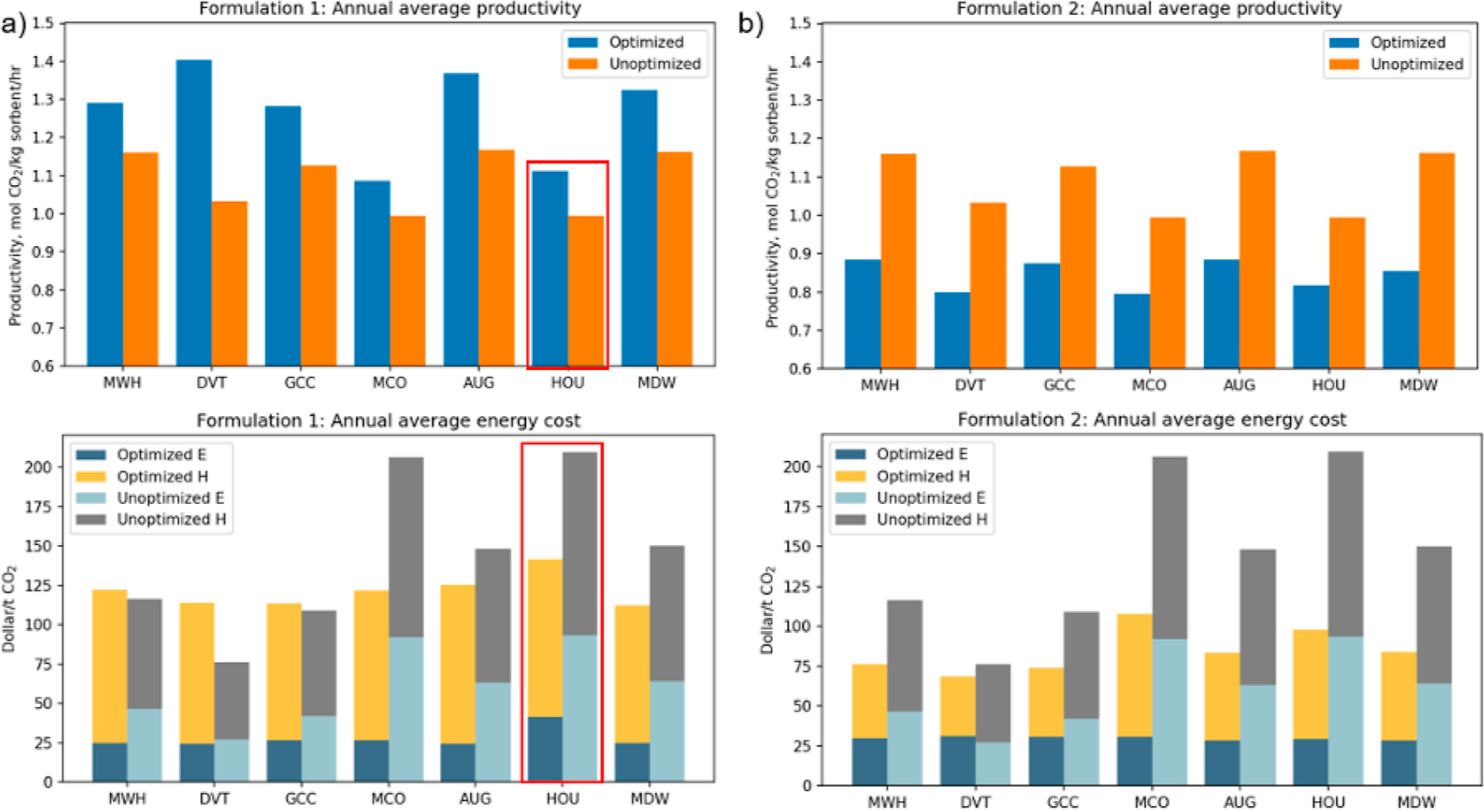
Optimization results of annual productivity and energy cost for the simulated DAC process (a) formulation 1 (productivity maximized) and (b) formulation 2 (energy cost minimized) for seven selected locations.

At each location, optimized cycle times were determined on a monthly basis using monthly average meteorological data from the lowest atmospheric layer of the CarbonTracker 1 × 1° gridded data. Annual averaged productivity and energy costs for 2018 are reported for the two optimization formulations for the selected locations in Figure 6. The “unoptimized” results in Figure 6 denote the results from the high-throughput results discussed above with fixed cycle times. Monthly optimized results of productivity and energy costs as well as the optimized cycle times can be found in Supporting Information, Figures S12–S18 and Tables S8–S14. Our optimization calculations for HOU in formulation 1 failed to find any feasible solutions in June and August for the input meteorological conditions, so the results in Figure 6 used the fixed cycle times from our earlier calculations for these months. The optimized results show some of the same seasonal trends as in the results from fixed cycle times (cf. Figures S6 and S16, for example). In the AUG location (Augusta, Maine), the optimized monthly productivity from formulation 1 varies from less than 1.2 to more than 1.6 mol CO_2_/kg sorbent/h, all of which are higher than the largest values observed anywhere in the CONUS using fixed cycle times (see Figure 3). Large temporal variation in the performance of this kind can have important implications for detailed process design. Detailed design of this kind would be needed to address issues such as potential increases in capital costs and complexity associated with processes that are not operated at a single steady state, for example.

The variation in annual average productivity among the optimized processes in formulation 1 is approximately 25%. Two of the locations with the best optimized results in this formulation are consistent with Figure 3, but the highest performing location is in Arizona, which is a low-performing region in Figure 3. This observation again highlights the need to adapt the details of specific DAC processes to local conditions. Previous modeling of similar adsorption-based DAC processes by Wiegner et al.^11^ suggested that in general low temperatures and high RH was beneficial for CO_2_ productivity. Our optimization results, however, suggest that the trade-offs that exist between different factors make it difficult to point to a single set of conditions as ideal for the specific DAC process we considered. In our results, the DVT location gives the highest productivity in part because the low RH allows shorter cycle times, but the AUG location has comparable productivity because the higher RH promotes coadsorption of CO_2_ and the lower average temperatures lead to temperature cycling over a wider range (in our process model).

There are strong differences in results between Formulation 1 (which optimized for productivity) and Formulation 2 (which optimized for energy costs) in Figure 6, with productivities 17–26% lower in the latter case. The optimized cycle times are considerably shorter in formulation 1 than in formulation 2 (Figures S12–S18). Purity of product CO_2_ was constrained to be over 95% for both formulations assuming that all water is condensed out. Detailed process optimization for DAC or similar processes must be considered on a multiobjective basis, and the process conditions chosen for practical operations are likely to be a trade-off among different objectives.

The process model we used for the specific adsorption-based process we considered, like any model of this kind, is based on a variety of input variables. Because data assessing process performance for a diverse range of ambient conditions (temperature, pressure, etc.) is sparse, information for various input variables must in some cases result from extrapolation beyond direct experimental data. Since our aim in this paper was to illustrate that variations in ambient conditions may have far-reaching implications for many DAC processes, we have not attempted to perform a sensitivity analysis on the thermodynamic and kinetic parameters in the specific process model we examined.

## Conclusions

Rapid implementation of the direct air capture of CO_2_ at the immense scales that will be needed to address global decarbonization goals will only be possible if highly efficient DAC processes are developed. This paper introduces a framework for assessing the impact of several previously underappreciated meteorological variables on the efficiency of DAC processes, including spatial and temporal variations in the quantities of CO_2_ available in ambient air. While our results are for a specific adsorption-based DAC process, this optimization analysis points to two issues that seem relevant for any effort to develop a large-scale DAC technology. First, optimization of process parameters in response to geographic and temporal variations in atmospheric conditions can have a large impact on the process performance. We performed optimization based on monthly average meteorological conditions, but in more refined process development, this kind of optimization could be performed on shorter time intervals using daily or even hourly meteorological data. Such analysis could, for example, take advantage in the large diurnal variations that exist in many locations.^7^

Second, it would be valuable for the DAC technical community to adopt a set of exemplar conditions that are representative of meteorological conditions in many locations to accelerate testing of promising technologies and facilitate meaningful comparisons between different studies.^34^ The meteorological data associated with the locations highlighted in Figure 5 and the nominal conditions listed in Table S5 offer examples of potential exemplar conditions, but selection of these conditions is best driven by consensus in the research community rather than by choices from a single research group.

The selection of sites for large-scale deployment of DAC is a multifaceted challenge. In addition to the performance of an installation from an engineering point of view, factors including access to infrastructure for disposition of the captured CO_2_, site permitting, community impacts, and community acceptance must be considered, as described in the so-called adoption readiness levels associated with deployment of new energy technologies.^6^ Resources that assess proximity to capacity for CO_2_ sequestration and access to purpose-built renewable energy resources for DAC at county-level resolution in the US are now available^35^ and information on this kind could be combined with the factors we have considered in this paper. The urgency of implementing carbon dioxide removal approaches and the significant capital that will be required to do so at scale mean that the development of DAC must address the full spectrum of these challenges simultaneously. It is hoped that the framework we have introduced in this paper will become a standard tool in assessing potential impacts of specific DAC processes and comparing these processes to other carbon dioxide removal and decarbonization strategies.

## Methods

### Humid Process Model

Elfving et al. used experimental adsorption data to develop a coadsorption mass-transfer model for CO_2_ and H_2_O using a proprietary amine-functionalized resin as a function of temperature and relative humidity as summarized below.^28^ For H_2_O adsorption mass transfer in the sorbent pore phase, the Guggenheim–Anderson–de Boer (GAB) isotherm from Gebald et al.^29^ and a linear driving force model was used
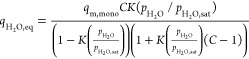
1
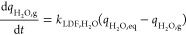
2Here,  is the H_2_O adsorption capacity at partial pressure of ,  is the saturation vapor pressure of H_2_O at the specified temperature, and *q*_m,mono_ is the monolayer saturation loading for H_2_O. *C* and *K* are temperature-dependent adsorption affinities defined in the Supporting Information. The linear driving force mass-transfer coefficient of H_2_O, , was fixed at 0.22 s^–1^ following Elfving et al. For the adsorption kinetics of CO_2_, Elfving et al.^14^ developed two separate adsorption-reaction mechanisms to account for dry and humid reactions. The CO_2_ adsorption-reaction model consists of two rate equations
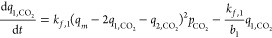
3
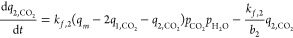
4

Equation 3 describes the dry reaction and eq 4 describes the humid reaction. *q*_*m*_ represents the total available amine sites for the adsorption of CO_2_ in the adsorbent. The forward reaction kinetic constants for dry and humid reactions are defined as *k*_*f*,1_ and *k*_*f*,2_, respectively. The backward reaction kinetic constants are written in terms of the adsorption affinities as *k*_*f*,*i*_/*b*_*i*_, with the adsorption affinity, *b*_*i*_, assumed to have a temperature-dependent form
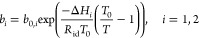
5Here, *b*_0,*i*_ is the reference adsorption affinity at *T*_0_ and *H*_*i*_ are the isosteric heats of adsorption for each reaction. eqs 3 and 4 are added together to obtain the total adsorption rate of CO_2_. In this model, the rate-limiting step was assumed to be the internal mass transfer of both humid and dry adsorption, and any influence of temperature on gas phase mass transfer was not considered.

We developed a process model based on Elfving et al.’s work to include the adsorption and the desorption cycles. A packed bed with the same geometry parameters and temperature vacuum swing adsorption (TVSA) process as in Elfving et al.^14,28^ was used. The same set of governing mass-transfer and heat-transfer equations that describes the cyclic process in that work was adopted here. As a TVSA cycle was modeled in this work, the Ergun equation was used to model the vacuum-induced gas velocity during desorption to simplify calculation of the pressure drop of the packed bed.^36^ Alternative models for pressure drop across the contactor would be needed if a process used structured contactors.^37^ The accuracy of this overall approach in evaluating the carbon capture performance of a cyclic TVSA process has been validated in several previous studies.^17,22,38−40^

All of the other process modeling equations, boundary conditions, and assumptions used in this work are listed in the Supporting Information. The values of the five parameters , *k*_*f*,1_, *k*_*f*,2_, *K*_*z*_, and *h* were taken from Elfving et al., which were based on experimental kinetic data from adsorption. The forward reaction rates, *k*_*f*,1_ and *k*_*f*,2_, were given by Elfving et al. at five relative humidity (RH) values in the range of 6–65%. Thus, these reaction constants were interpolated from those values at relative humidities inside this range. Outside of this range, both reaction constants are set to be equal to the rate constants at RH of 6 or 65%. The axial effective heat conductivity, *K*_*z*_, and overall heat-transfer coefficients, *h*, are fixed using the 2 vol % data H_2_O at 25 °C from Elfving et al.^14^ The differential equations defining this process model were solved using discretization along the axial direction for differential variables in gPROMS.^41^ For input conditions where the solver failed to find a solution, the number of discretization steps was increased until a stable solution was found. An example of the results from gPROMS being used to obtain cyclic steady-state information is given in Supporting Information, Figure S1.

### High-Throughput Data Collection

We considered both electrical energy and heat in assessing the requirements of the DAC process that we modeled. Electrical energy inputs were calculated using fan blower energy during the adsorption and the vacuum pump energy during the desorption step. To describe a scaled-up DAC unit, the blower energy reported by Sabatino et al.^27^ was multiplied by a factor of 27.78. The vacuum pump energy was scaled after assuming that all the water will be condensed out before reaching the vacuum pump. The heat energy requirement calculations were adapted from previous works of Sinha et al.^22^ and Sabatino et al.,^27^ which separated the sensible heats of CO_2_, H_2_O, and sorbent and adsorption reaction heats of CO_2_ and H_2_O. Sensible heat calculations were performed by accounting for the need to heat the system during the sorbent regeneration, while adsorption heats were estimated using isosteric heats of adsorption for CO_2_ and H_2_O. All energy requirements are reported normalized to the CO_2_ amount in the product stream. Values of these metrics were calculated as the outputs from gPROMS, with further details given in the Supporting Information.

To choose a reasonable set of operating parameters to simulate at the 25,344 input conditions, as listed in Table S5, we first developed an optimized set of operating parameters at standard ambient conditions (*T*_amb_ = 298 K, *P*_amb_ = 105 kPa,  = 400 ppm) at 60% RH. This optimization used a python data-driven surrogate-based branch-and-bound (DDSBB) optimization package developed by Zhai and Boukouvala.^42,43^ This approach treats gPROMS simulations as a black-box function and fits the output with surrogate models as well as convex underestimates to determine the global optimum. Information on the uniform sampling inputs used with gPROMs is available as a supplementary file, and further information about the DDSBB calculations is given in the Supporting Information. The optimal operating parameters at ambient conditions were then used to simulate the DAC performance at the full range of meteorological conditions (*T*_amb_, *P*_amb_,  and RH) in Table S5 using gPROMS. A detailed table of input parameters for the process model is given in the Supporting Information.

## Supporting Information Available

gPROMS process model, degrees of freedom and applied in the process optimization and the optimized values, TVSA process model, meteorological parameters sampling space, 2018 year averages of lowest atmospheric layer meteorology, grid cell year average partial pressure, energy requirements and productivity, monthly average meteorological trends, optimized productivity, energy costs, as well as cycle times, and meteorological inputs and optimized productivity, energy costs, as well as cycle times (PDF)
Parameter details in CSV format (ZIP)
