# Supplementary material for: Assessing Impacts of Atmospheric Conditions on Efficiency and Siting of Large-Scale Direct Air Capture Facilities
Source: JACS Au. 2024 May 1;4(5):1883–91. doi: 10.1021/jacsau.4c00082 (PMC11134380; doi:10.1021/jacsau.4c00082)
Supplement: Supplementary file 1 — au4c00082_si_001.pdf [file au4c00082_si_001.pdf]

# Supporting Information for

## Assessing Impacts of Atmospheric Conditions on Efficiency and Siting of Large-scale Direct Air Capture Facilities

Xuqing Cai,[‡] Mark A. Coletti,[¶] David S. Sholl,[¶,‡] and Melissa R. Allen-Dumas[*,¶]

[‡]School of Chemical & Biomolecular Engineering, Georgia Institute of Technology, Atlanta, GA 30332, USA

[¶]Oak Ridge National Laboratory, 1 Bethel Valley Rd. Oak Ridge, TN 37831, USA

*Email: allenmr@ornl.gov

**This PDF file includes:**

Supporting text

Figures S1 to S18

Tables S1 to S14

SI References

## Other Supporting Data

In addition to this pdf file, four additional data files are available as Supporting Information within a zip file. These four files are:

**CarbonTracker2018_inter.csv:** Monthly CarbonTracker Data of 2018 inputs including interpolated performance values

**Form1.csv:** Monthly performance data from optimization formulation 1 (maximizing productivity) for 7 locations

**Form2.csv:** Monthly performance data from optimization formulation 2 (minimizing cost) for 7 locations

**UniformSample.csv:** Uniform sampling point inputs and calculations from gPROMS process modeling

## Model Assumptions For Packed Bed TVSA

- Ideal gas law was assumed for all gas species.
- Radial gradients were neglected, including dispersion and heat conduction.
- $N_2$ was treated as a non-adsorbing component in the gas.
- The void fraction in the bed was considered uniform in the packed bed.
- Pressure drop along the axial-direction was described by the Ergun equation.
- Mass transfer was assumed to be limited by adsorption-reaction.
- The linear driving-force (LDF) approximation describes the mass transfer between gas-phase and sorbent-phase.
- Gas-phase and sorbent-phase temperature was assumed to be equal.
- The axial gradient of indirect cooling/heating water outside of the column wall was neglected; the water temperature was only assumed to be time-dependent.

## Model Equations

Temperature-dependent adsorption affinity terms C and K in GAB isotherm:

$$C = C_0 \exp(\frac{\Delta H_C}{R_{id}T})$$

$$K = K_0 \exp(\frac{\Delta H_K}{R_{id}T})$$

Component mass balance equations (gas-phase):

$$\frac{\partial C_i}{\partial t} = D_L \frac{\partial^2 C_i}{\partial z^2} - v_i \frac{\partial C_i}{\partial z} - \frac{\rho_B}{\varepsilon} \frac{\partial q_i}{\partial t}$$

where $q_i$ for $H_2O$ and $CO_2$ were given in Equation 1-3 from the main text

Momentum balance equation (Ergun Equation):

$$-\frac{\partial P_{tot}}{\partial z} = 150 \frac{\mu_g (1-\varepsilon)^2}{\varepsilon^3 (2R_p)^2} u_g(z) + 1.75 \frac{(1-\varepsilon)\rho_g}{2R_p \varepsilon^3} |u_g(z)| u_g(z)$$

Energy balance equation:

$$\frac{1-\varepsilon}{\varepsilon}\rho_p\left(C_{p,a}+C_{p,CO2}\cdot MW_{CO2}\cdot q_{tot,CO2}+C_{p,H2O}\cdot MW_{H2O}\cdot q_{H2O}\right)\frac{\partial T}{\partial t}$$
$$=\frac{K_z}{\varepsilon}\frac{\partial^2 T}{\partial z^2}-\rho_g C_{p,g} u_g(z)\frac{\partial T}{\partial z}-\frac{1-\varepsilon}{\varepsilon}\rho_p\left(\Delta H_1\frac{\partial q_{1,CO2}}{\partial t}+\Delta H_2\frac{\partial q_{2,CO2}}{\partial t}+\Delta H_{H2O}\frac{\partial q_{H2O}}{\partial t}\right)$$
$$-\frac{2h}{\varepsilon R_{bed}}(T-T_w)$$

Boundary Conditions that change with steps in the TVSA process illustrated in Figure S1:

| z = 0 | z = L |
|---|---|
| **Adsorption** | |
| $u_g(z=0)=u_{ads}$ | $\frac{\partial u_g(z=L)}{\partial z}=0$ |
| $\frac{\partial P_{tot}(z=0)}{\partial z}=0$ | $P_{tot}(z=L)=P_{atm}$ |
| $T_w=T_{w,ads}$ | |
| **Vacuum 1** | |
| $u_g(z=0)=\frac{u_{ads}-u_{des}}{2}\tanh\left(\frac{t_{vac1}-t}{2}\right)+\frac{u_{ads}+u_{des}}{2}$ | $\frac{\partial u_g(z=L)}{\partial z}=0$ |
| $\frac{\partial P_{tot}(z=0)}{\partial z}=0$ | $P_{tot}(z=L)=P_{atm}$ |
| $T_w=T_{w,ads}$ | |
| **Vacuum 2** | |
| $u_g(z=0)=u_{des}$ | $\frac{\partial u_g(z=L)}{\partial z}=0$ |
| $\frac{\partial P_{tot}(z=0)}{\partial z}=0$ | $P_{tot}(z=L)=\frac{P_{ads}-P_{des}}{2}\tanh\left(\frac{t_{vac2}-t}{2}\right)+\frac{P_{ads}+P_{des}}{2}$ |
| $T_w=T_{w,ads}$ | |
| **Heating** | |
| $u_g(z=0)=u_{des}$ | $\frac{\partial u_g(z=L)}{\partial z}=0$ |
| $\frac{\partial P_{tot}(z=0)}{\partial z}=0$ | $P_{tot}(z=L)=P_{des}$ |
| $\frac{\partial T_w}{\partial t}=\frac{T_{w,des}-T_{w,ads}}{t_{heat}}$ | |
| **Desorption/Regeneration** | |
| $u_g(z=0)=u_{des}$ | $\frac{\partial u_g(z=L)}{\partial z}=0$ |
| $\frac{\partial P_{tot}(z=0)}{\partial z}=0$ | $P_{tot}(z=L)=P_{des}$ |
| $T_w=T_{w,des}$ | |
| **Cooling** | |
| $u_g(z=0)=u_{des}$ | $\frac{\partial u_g(z=L)}{\partial z}=0$ |
| $\frac{\partial P_{tot}(z=0)}{\partial z}=0$ | $P_{tot}(z=L)=P_{des}$ |
| $\frac{\partial T_w}{\partial t}=\frac{T_{w,ads}-T_{w,des}}{t_{cool}}$ | |
| **Pressurization 1** | |
| $u_g(z=0)=u_{des}$ | $\frac{\partial u_g(z=L)}{\partial z}=0$ |
| $\frac{\partial P_{tot}(z=0)}{\partial z}=0$ | $P_{tot}(z=L)=\frac{P_{ads}-P_{des}}{2}\tanh\left(\frac{t-t_{press1}}{2}\right)+\frac{P_{ads}+P_{des}}{2}$ |

| |
|---|
| $T_w = T_{w,ads}$ |

| Pressurization 2 | |
|---|---|
| $u_g(z=0) = \frac{u_{ads} - u_{des}}{2}\tanh\left(\frac{t - t_{press2}}{2}\right) + \frac{u_{ads} + u_{des}}{2}$ <br> $\frac{\partial P_{tot}(z=0)}{\partial z} = 0$ | $\frac{\partial u_g(z=L)}{\partial z} = 0$ <br> $P_{tot}(z=L) = P_{atm}$ |
| $T_w = T_{w,ads}$ | |

Other constant boundary conditions:

| z = 0 | z = L |
|---|---|
| $D_L \frac{\partial C_i(z=0)}{\partial z} = -u_g(C_{i,feed} - C_i(z=0))$ | $\frac{\partial C_i(z=L)}{\partial z} = 0$ |
| $K_z \frac{\partial T(z=0)}{\partial z} = -\varepsilon u_g \rho_g C_{p,g}(T_{feed} - T(z=0))$ | $\frac{\partial T(z=L)}{\partial z} = 0$ |

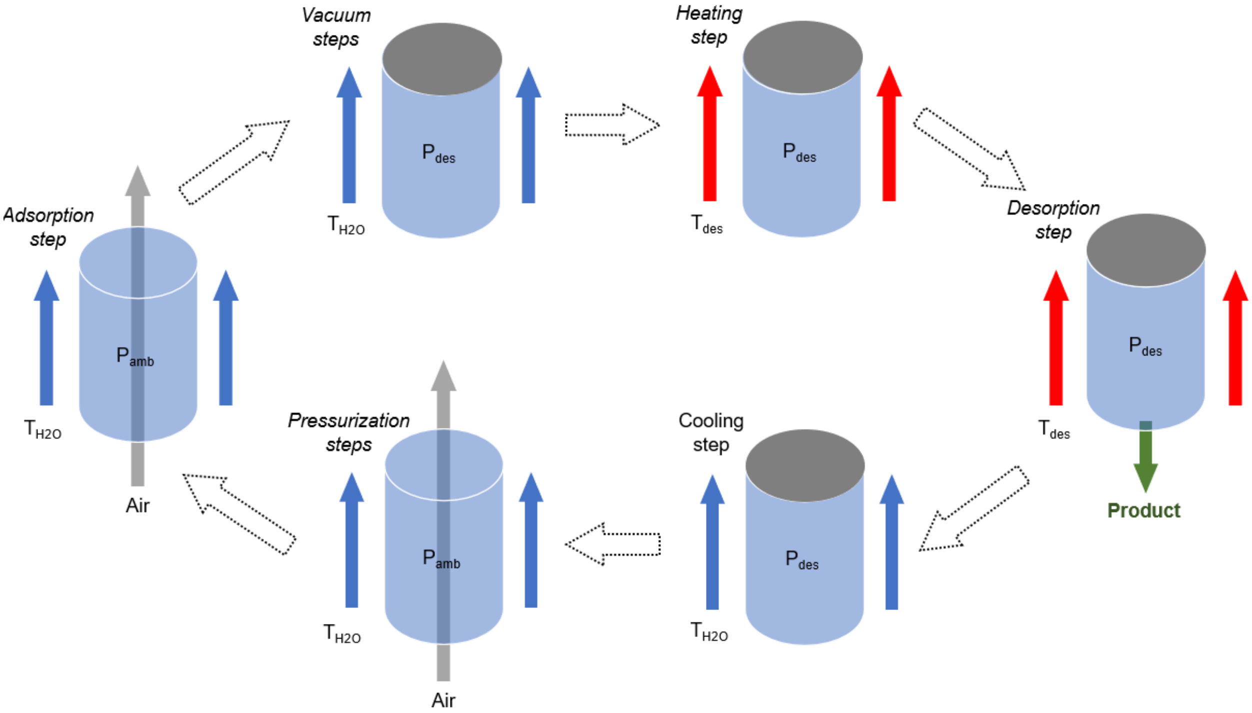


Figure S1. Illustration of the TVSA process used for gPROMS process simulations.

Electrical energy requirements for blower:

$$E_{blow} = 27.78 \cdot \frac{\Delta P_{tot}\dot{Q}_{ads}t_{ads}}{N_{CO2,\,prod}}$$

To resemble a scaled-up DAC unit, a coefficient of 27.78 was multiplied to the blower energy by assuming the inlet gas velocity to be 2.9 m/s and the pressure drop to be 100 Pa along the contactor.

Electrical energy requirements for vacuum pump:

$$E_{vac} = \frac{N_{prod} - N_{H2O,\,prod}}{N_{prod}} \frac{1}{N_{CO2,\,prod}} \frac{1}{\eta_{vac}} \frac{\gamma}{1-\gamma} A \int_{t_{vac2}}^{t_{des}} u_g(L)\, P_{tot}(L) \left[ \left( \frac{P_{ads}}{P_{tot}(L)} \right)^{\frac{\gamma-1}{\gamma}} - 1 \right] dt$$

Vacuum pump energy was scaled by the ratio of all product stream excluding water vapor and the total product stream. This assumes that all the water is condensed out before the reaching the vacuum pump.

$CO_2$ sensible heat:

$$H_{CO2,sens} = \frac{m_{sorbent} C_{p,CO2}}{L N_{CO2,\,prod}} \int_{t_{vac2}}^{t_{des}} \int_0^L \frac{\partial T(z)}{\partial t} q_{CO2,tot}\,(z)\, dzdt$$

$H_2O$ sensible heat:

$$H_{H2O,sens} = \frac{m_{sorbent} C_{p,H2O}}{L N_{CO2,\,prod}} \int_{t_{vac2}}^{t_{des}} \int_0^L \frac{\partial T(z)}{\partial t} Q_{H2O}(z)\, dzdt$$

Sorbent sensible heat:

$$H_{sorbent,sens} = \frac{m_{sorbent} C_{p,a}}{L N_{CO2,\,prod}} \int_{t_{vac2}}^{t_{des}} \int_0^L \frac{\partial T(z)}{\partial t} dzdt$$

$CO_2$ adsorption heat:

$$H_{CO2,ads} = \frac{m_{sorbent}}{L N_{CO2,\,prod}} \int_{t_{vac2}}^{t_{des}} \int_0^L (q_{CO2,1}(z)\Delta H_1 + q_{CO2,2}(z)\Delta H_2)\, dzdt$$

$H_2O$ adsorption heat:

$$H_{H2O,ads} = \Delta H_{H2O} \frac{N_{H2O,\,prod}}{N_{CO2,\,prod}}$$

Mass of sorbent:

$$m_{sorbent} = (1-\varepsilon)\rho_p \pi R_{bed}^2$$

## Model inputs and Example Calculations

All input parameters to gPROMS process are listed in Table S1-Table S*3*. The temperature of cooling water, $T_{H2O}$, during the adsorption step was set to be the same as the ambient temperature with lower bound of 278 K and an upper bound of 303 K of the cooling water temperature. If the ambient temperature fell outside of this range, the lower bound or the upper bound water temperature was used. Table S4 contains the variables calculated from gRPOMS, while Table S*5* describes the meteorological inputs for high throughput grid-sampling data calculation. DDSBB error tolerances were set to be 0.05 with initial sample size of 20 gPROMS calculations. A total of 300 calculations was selected for the sample limits for one optimization iteration. The equal bisection method was used for the branch and bound operations and the neural network with the package's default settings was used for the multifidelity model. Table S1 indicates that the number of cycle times is set to 4 for all samples. The reason for this choice is that preliminary tests indicated that using four cycles typically gives differences of performance metrics (productivity, recovery, heat and electricity requirements) to within 1% between the final two cycles, so four cycles was used as giving a good approximation of a cyclic steady state.

Table S1. Input parameters to gPROMS process model for high-throughput data sampling

| **Meaning** | **Parameter** | **Unit** | **Data Sources** |
|---|---|---|---|
| Adsorption time | $t_{ads}$ | s | Optimized from DDSBB |
| Desorption time | $t_{des}$ | s | Optimized from DDSBB |
| Cycle number | No_cycle | -- | Set to 4 for all samples |
| Dry reaction rate constant | $k_{f,1}$ | $bar^{-1}\ s^{-1}\ (mol/kg)^{-1}$ | Interpolated using RH |
| Humid reaction rate constant | $k_{f,2}$ | $bar^{2}\ s^{-1}$ | Interpolated using RH |
| LDF $H_2O$ mass transfer coefficient | $k_{H2O}$ | $s^{-1}$ | Elfving et al. |
| Axial effective heat conductivity | $K_z$ | W/m/K | Elfving et al. |
| Overall heat transfer coefficient | $h$ | $W/m^2/K$ | Elfving et al.[1] |
| Ambient $H_2O$ volume fraction | $y_{H2O}$ | -- | Sampling space, Table S5 |
| Ambient $CO_2$ volume fraction | $y_{CO2}$ | -- | Sampling space, Table S5 |
| Ambient temperature | $T_{feed}$ | K | Sampling space, Table S5 |
| Ambient Pressure | $P_{atm}$ | Pa | Sampling space, Table S5 |
| Cooling water temperature | $T_{w,\ ads}$ | K | See description above |

Table S2. Degrees of freedom and applied in the process optimization and the optimized values.

| **Parameter** | **Unit** | **Lower bound** | **Upper bound** | **Optimized Value** |
|---|---|---|---|---|

| | | | | |
|---|---|---|---|---|
| $t_{ads}$ | s | 100 | 2,000 | 1,725 |
| $t_{des}$ | s | 100 | 2,000 | 890 |

Table S3. Parameters applied in the TVSA process model.

| **Parameter** | **Symbol** | **Value** | **Unit** |
|---|---|---|---|
| Packed bed length | $L$ | 0.018 | m |
| Packed bed radius | $R_{bed}$ | 0.0045 | m |
| Sorbent pellets radius | $R_p$ | $3\times10^{-4}$ | m |
| Packed bed void fraction | $\varepsilon$ | 0.6 | -- |
| Packed bed bulk density | $\rho_B$ | 450 | $kg/m^3$ |
| Pellet density | $\rho_p$ | 720 | $kg/m^3$ |
| Sorbent pellets specific heat capacity | $C_{p,a}$ | 1580 | J/kg/K |
| $CO_2$ specific heat capacity | $C_{p,CO2}$ | 855 | J/kg/K |
| $H_2O$ specific heat capacity | $C_{p,H2O}$ | 4180 | J/kg/K |
| Gas mixture specific heat capacity | $C_{p,g}$ | 1055 | J/kg/K |
| GAB isotherm monolayer saturated $H_2O$ | $q_{m,mono}$ | 2.58 | mol/kg sorbent |
| GAB isotherm parameter | $C_0$ | 0.155 | -- |
| GAB isotherm parameter | $K_0$ | 0.871 | -- |
| GAB isotherm isosteric heat parameter | $\Delta H_C$ | 6.6 | kJ/mol |
| GAB isotherm isosteric heat parameter | $\Delta H_K$ | 0 | kJ/mol |
| $H_2O$ sorption isosteric heat | $\Delta H_{H2O}$ | 50.7 | kJ/mol |
| Ideal gas constant | $R$ | 8.314 | J/mol/K |
| Total amine site for $CO_2$ sorption | $q_m$ | 1.91 | mol/kg sorbent |
| Dry $CO_2$ adsorption affinity at 298 K | $b_{01}$ | $3.199\times10^{-2}$ | $Pa^{-1}(mol/kg)^{-1}$ |
| Humid $CO_2$ adsorption affinity at 298 K | $b_{02}$ | $7.461\times10^{-6}$ | $Pa^{-2}$ |
| Dry $CO_2$ isosteric heat parameter | $\Delta H_1$ | 98 | kJ/mol |
| Humid $CO_2$ isosteric heat parameter | $\Delta H_2$ | 111 | kJ/mol |
| Standard temperature | $T_0$ | 298 | K |
| Gas viscosity | $\mu_g$ | $1.983\times10^{-5}$ | Pa*s |
| Gas density | $\rho_g$ | 1.15 | $kg/m^3$ |

| | | | |
|---|---|---|---|
| Axial dispersion coefficient | $D_L$ | $3.5\times10^{-4}$ | $m^2/s$ |
| Vacuum pump efficiency | $\eta_{vac}$ | 0.5 | |
| Adiabatic constant | $\gamma$ | 1.4 | -- |
| Feed gas velocity for adsorption step | $u_{ads}$ | 0.349 | m/s |
| Feed gas velocity for desorption step | $u_{des}$ | $10^{-10}$ | m/s |
| Feed gas volumetric flow rate | $\dot{Q}_{ads}$ | $2.22\times10^{-5}$ | $m^3/s$ |
| Desorption step vacuum pressure | $P_{des}$ | $10^4$ | Pa |
| Desorption step heating water temperature | $T_{des}$ | 373 | K |

Table S4. Other variables calculated from TVSA process model.

| **Variables** | **Symbol** | **Unit** |
|---|---|---|
| Mass of sorbent | $m_{sorbent}$ | kg |
| Dry $CO_2$ adsorbed amount | $q_{CO2,1}$ | mol/kg |
| Humid $CO_2$ adsorbed amount | $q_{CO2,2}$ | mol/kg |
| Total $CO_2$ adsorbed amount | $q_{CO2,tot}$ | mol/kg |
| $CO_2$ mass in product stream | $N_{CO2,\,prod}$ | tonne |
| $N_2$ mass in product stream | $N_{N2,\,prod}$ | tonne |
| $H_2O$ mass condensed from product | $N_{H2O,\,prod}$ | tonne |
| Heat requirements | $H$ | kWh/t $CO_2$ |
| Electrical energy requirements | $E$ | kWh/t $CO_2$ |
| $CO_2$ productivity | $Prod$ | mol $CO_2$/kg sorbent/hr |

Table S5. Meteorological parameters sampling space for high-throughput data collection (N = 25,344)

| | **Lower Bound** | **Upper Bound** | **# of Points** | **Sample Interval** |
|---|---|---|---|---|
| Pressure (Pa) | 70,000 | 105,000 | 8 | 5,000 |
| Relative Humidity (%) | 0 | 100 | 11 | 10 |
| $CO_2$ (ppm) | 380 | 465 | 18 | 5 |
| Temperature | 240 | 315 | 16 | 5 |

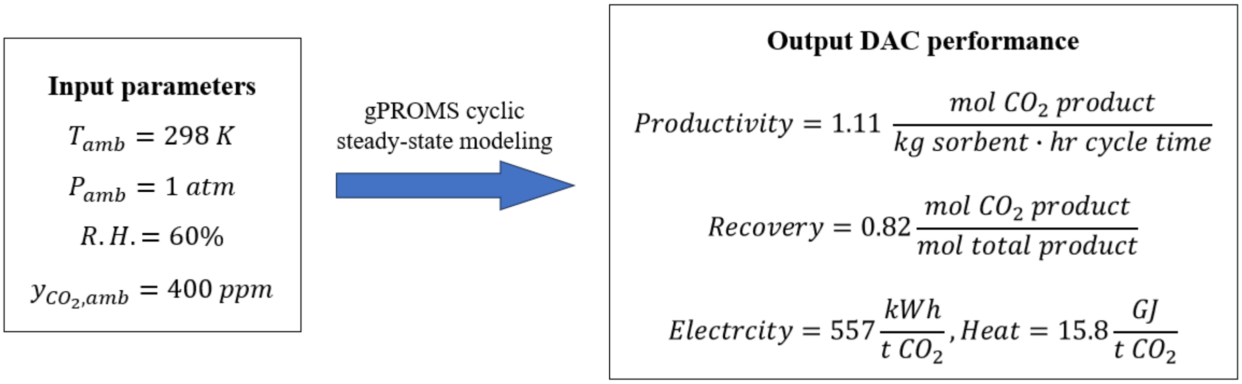


Figure S2. An example calculation using meteorological inputs to gPROMS to reach cyclic steady-state and the corresponding outputs using the optimized cycle time from Table S2.

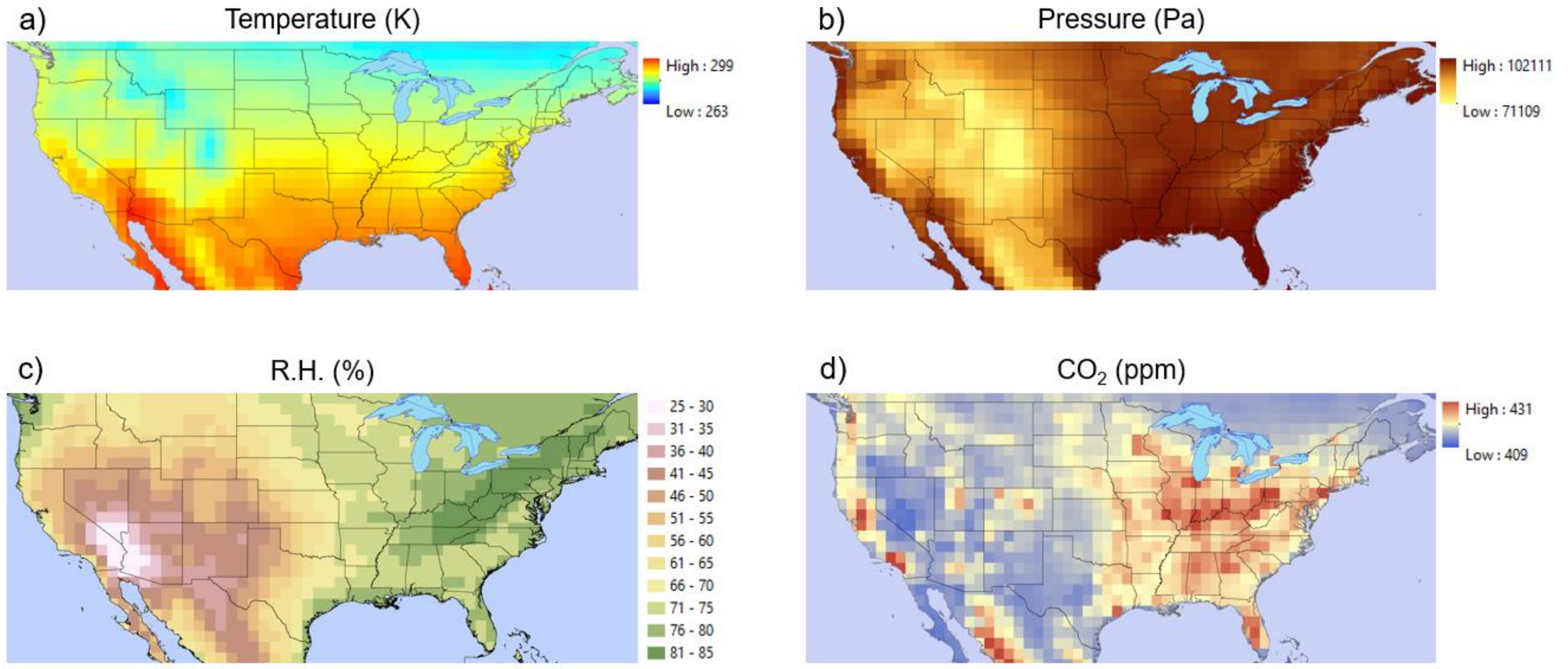


Figure S3. 2018 year averages of lowest atmospheric layer meteorology for each 1x1 degree grid cell across the continental US.[2] These values were used as input to the DAC process model.

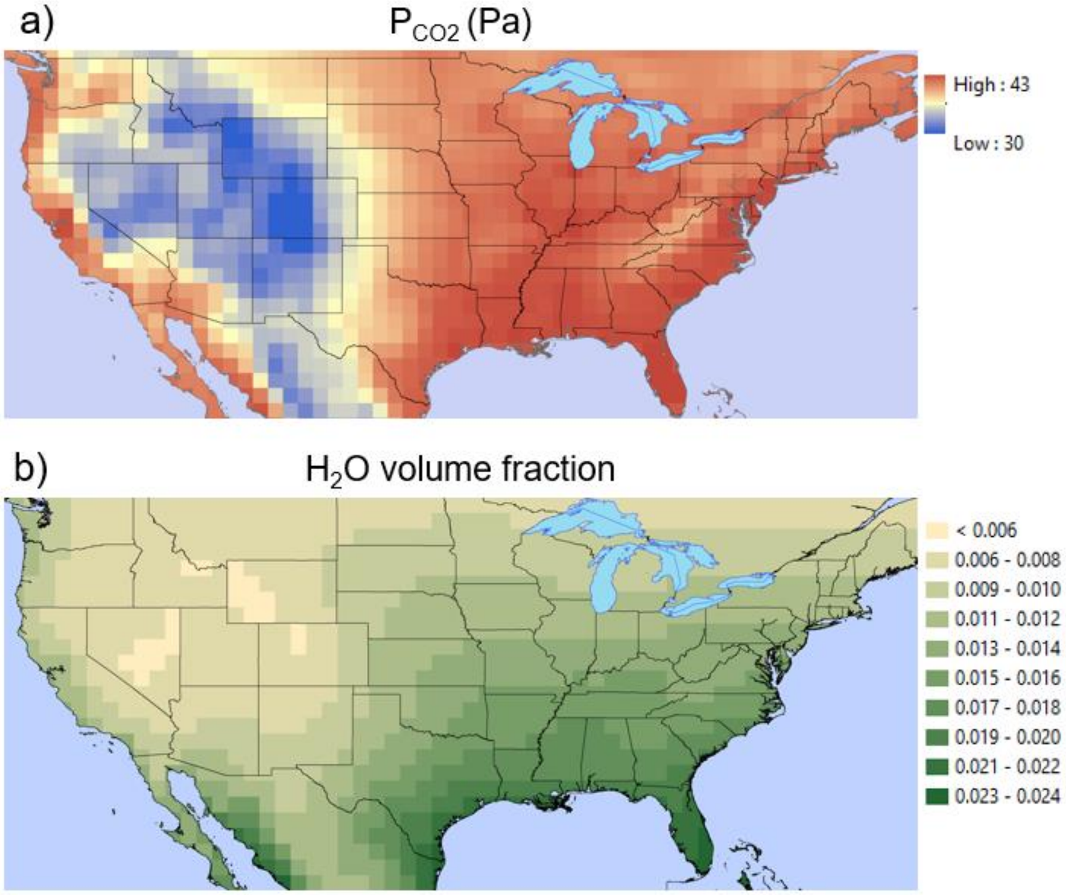


Figure S4. Grid cell year average partial pressure of $CO_2$ and $H_2O$ volume fraction for 2018.

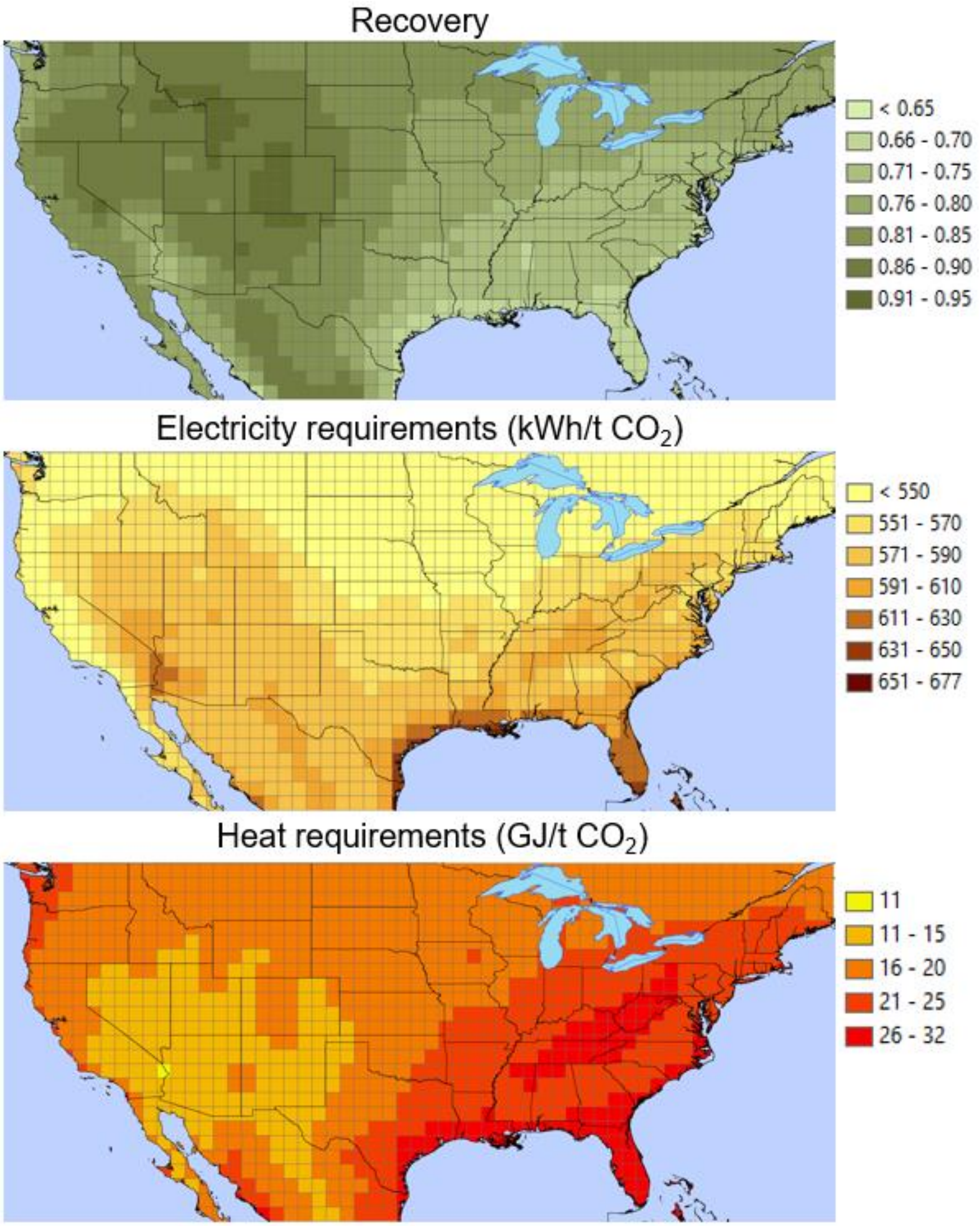


Figure S5. Annual average DAC performance over CONUS during 2018 from interpolation calculations using meteorological variables at 1x1° latitude/longitude grid spacing.

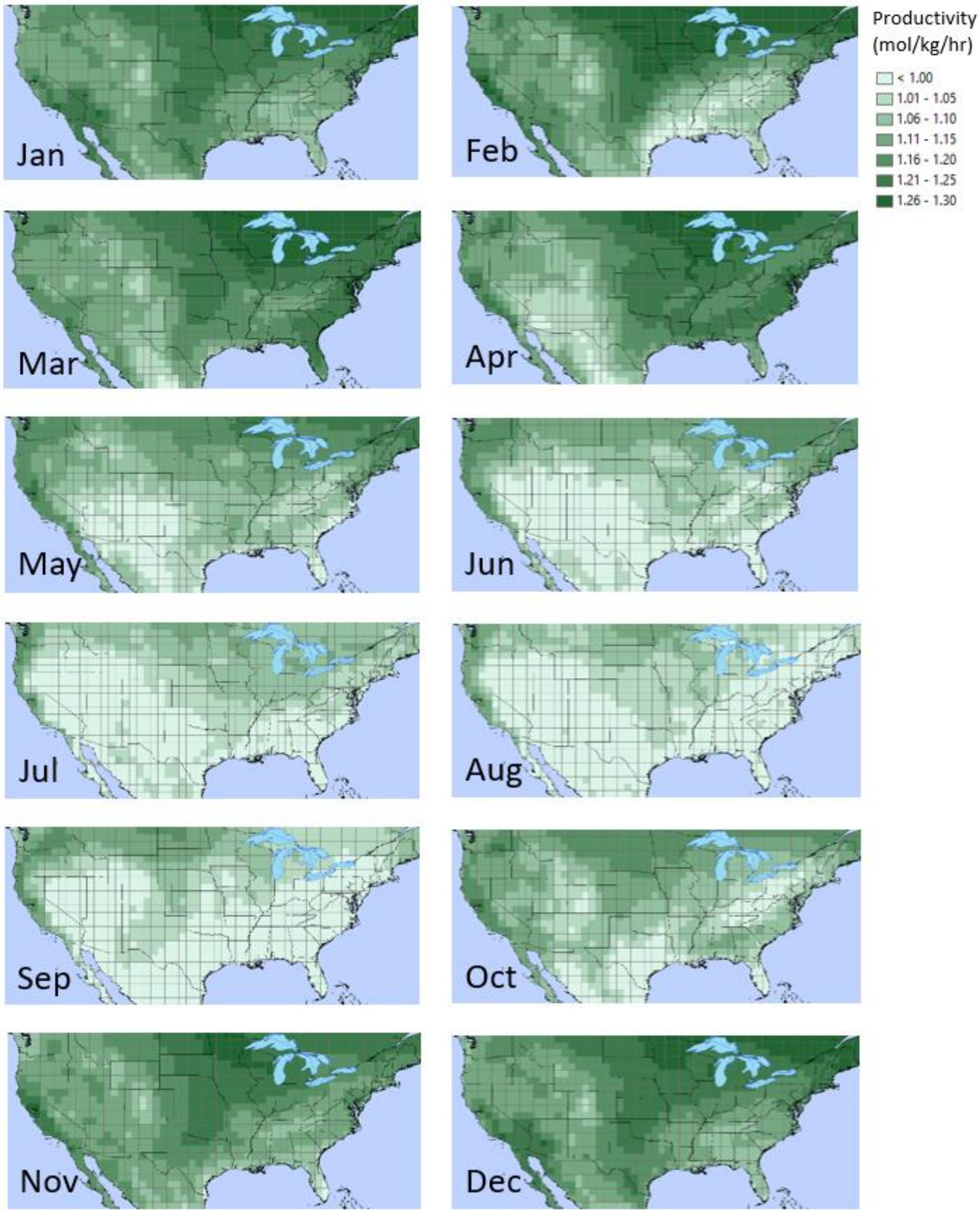


Figure S6. Monthly average productivity from high-throughput data collection.

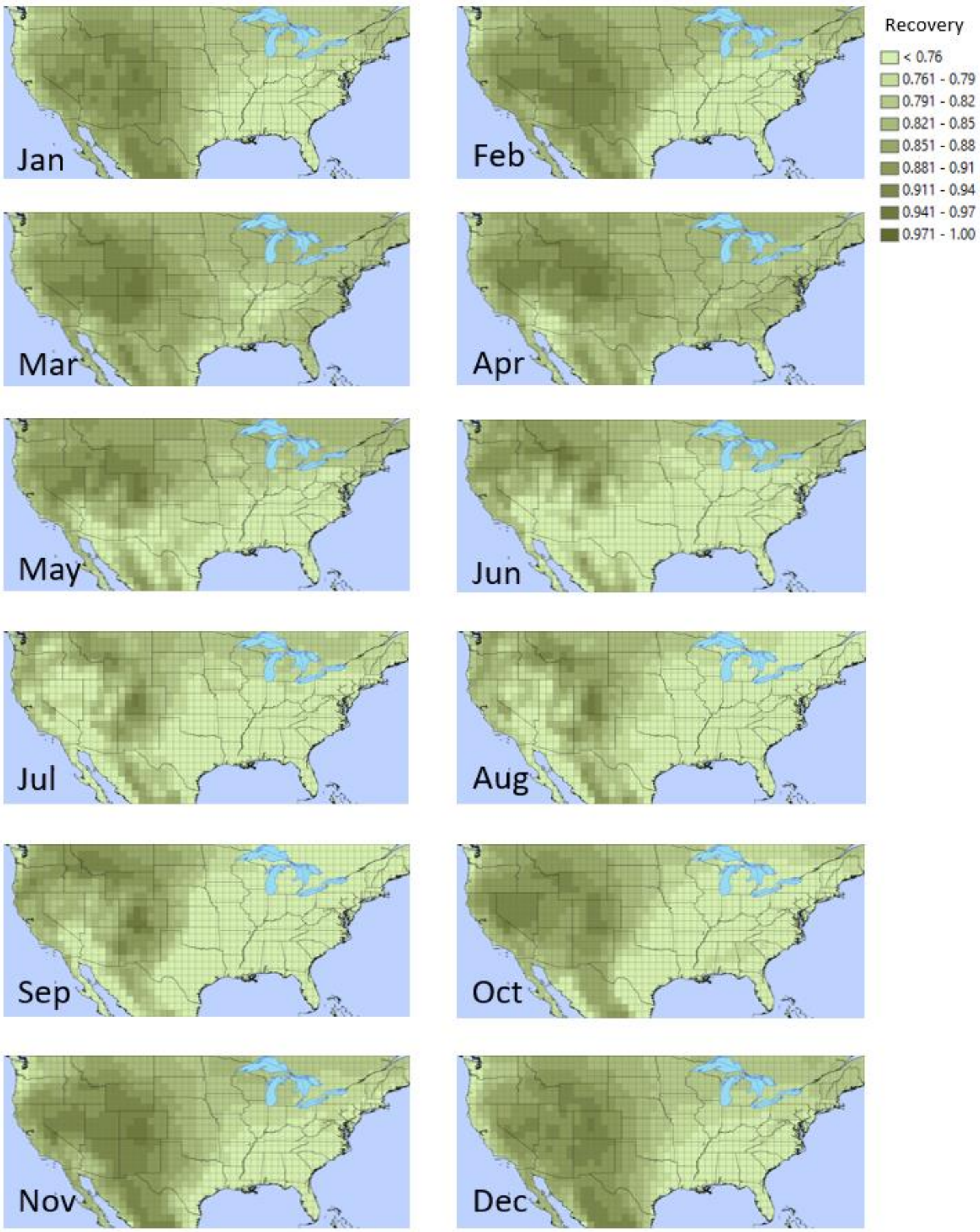


Figure S7. Monthly average recovery from high-throughput data collection

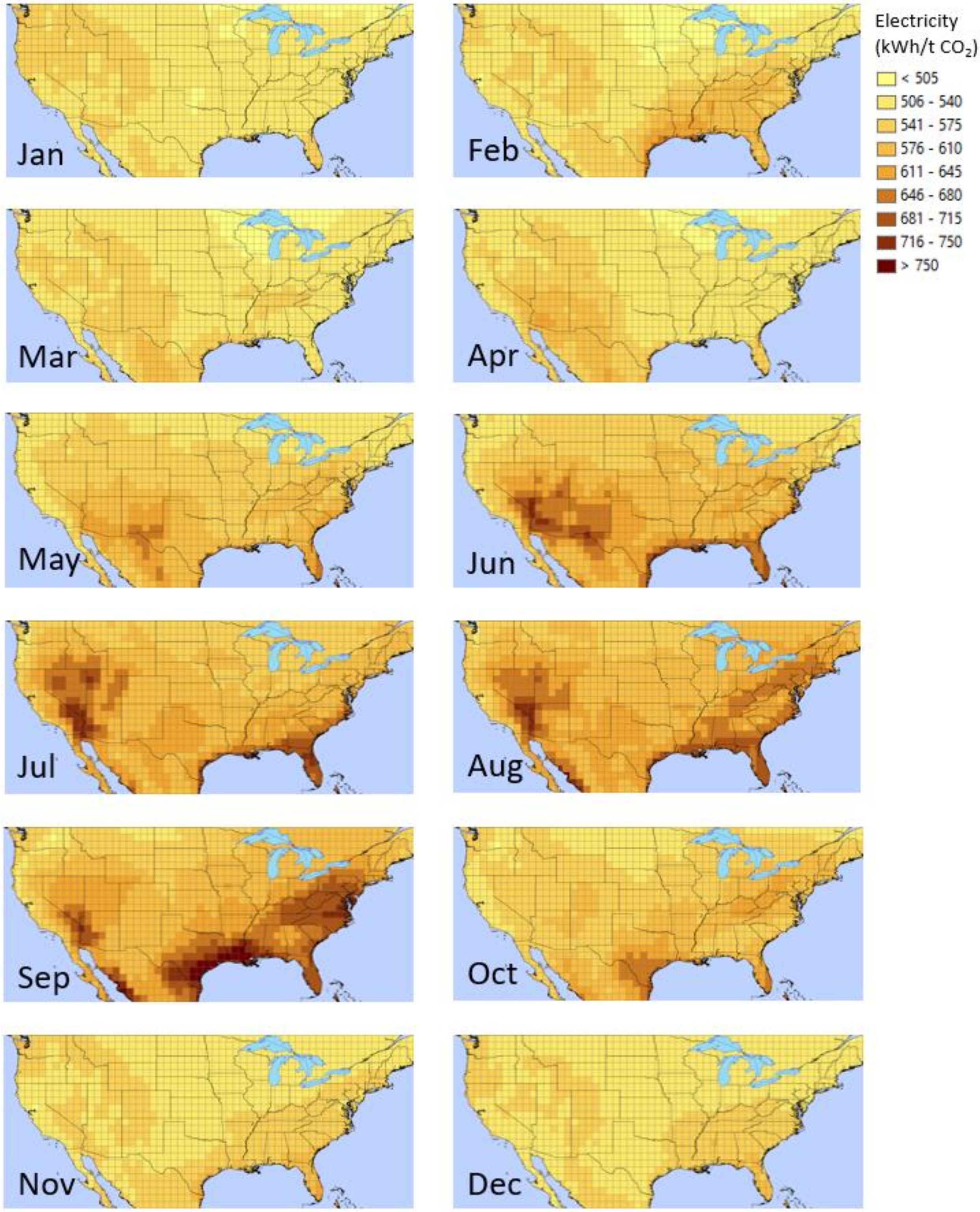


Figure S8. Monthly average electricity requirements from high-throughput data collection.

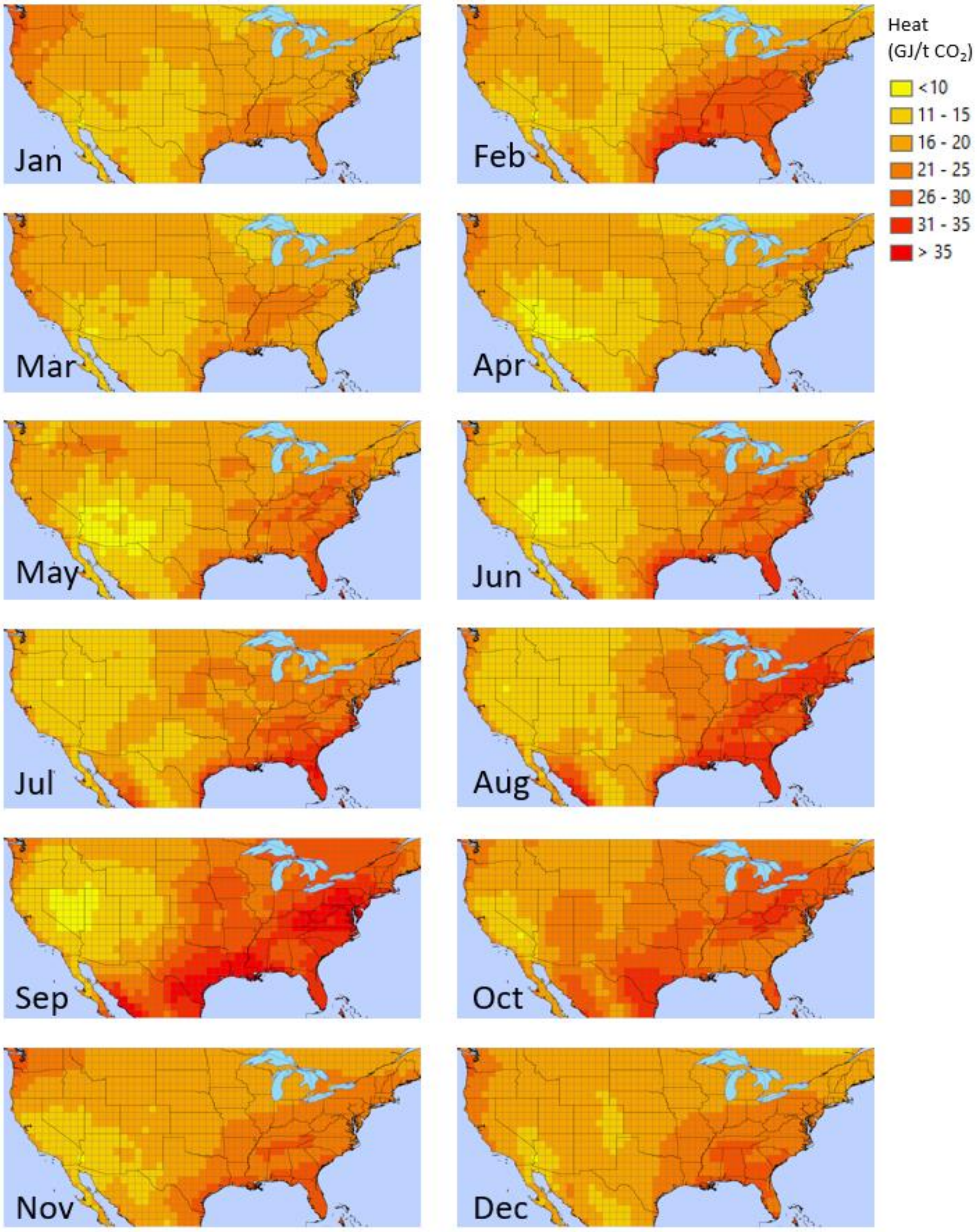


Figure S9. Monthly average heat requirements from high-throughput data collection.

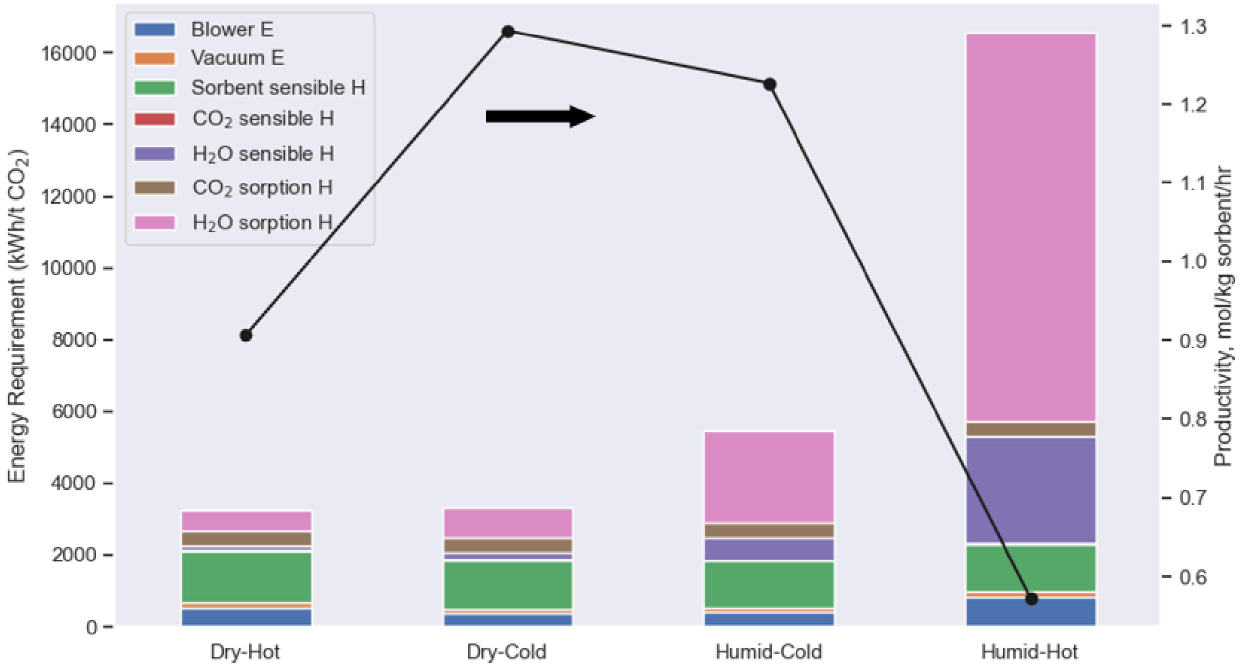


Figure S10. Energy requirements and productivity of at four designated ambient conditions listed in Table S6. Right axis is the productivity corresponding to the black line on the figure. Heat requirements were converted to kWh from GJ using 1 GJ = 277.78 kWh.

Table S6. Four designated ambient conditions for energy penalty case study.

| **Condition** | **$T_{amb}$ (K)** | **R.H. (%)** | **$CO_2$ (ppm)** | **$P_{amb}$ (Pa)** |
|---|---|---|---|---|
| Dry-Hot | 303 | 25 | | |
| Dry-Cold | 273 | 50 | 400 | $10^5$ |
| Humid-Hot | 303 | 95 | | |
| Humid-Cold | 273 | 95 | | |

Table S7. Detailed location information of the seven picked locations for process optimization

| States | Location | Condition | Code | Longitude | Latitude | Elevation (m) |
|---|---|---|---|---|---|---|
| AZ | Phoenix Deer Valley Airport | dry-hot | DVT | -112 | 34 | 450 |
| FL | Orlando International Airport | humid-hot | MCO | -81 | 28 | 32 |
| WY | Gillette–Campbell County Airport | dry-cold | GCC | -106 | 44 | 1230 |
| WA | Moses Lake Grant County | humid-cold | MWH | -119 | 47 | 361 |
| IL | Chicago-Midway Airport | Lake effect | MDW | -88 | 42 | 188 |
| ME | Augusta state Airport | Inland | AUG | -70 | 44 | 107 |
| TX | Houston Will Hobby Airport | Coastal | HOU | -95 | 30 | 14 |

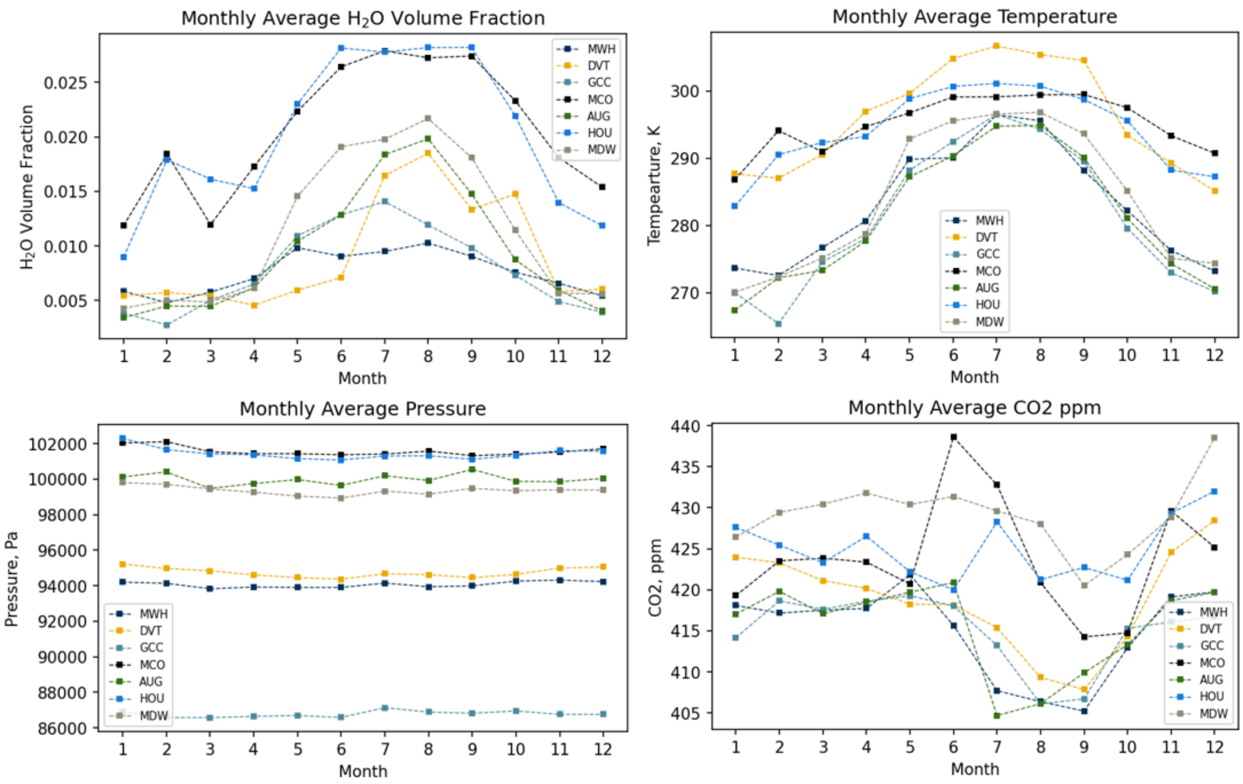


Figure S11. Monthly average meteorological trends of 2018 for the selected locations

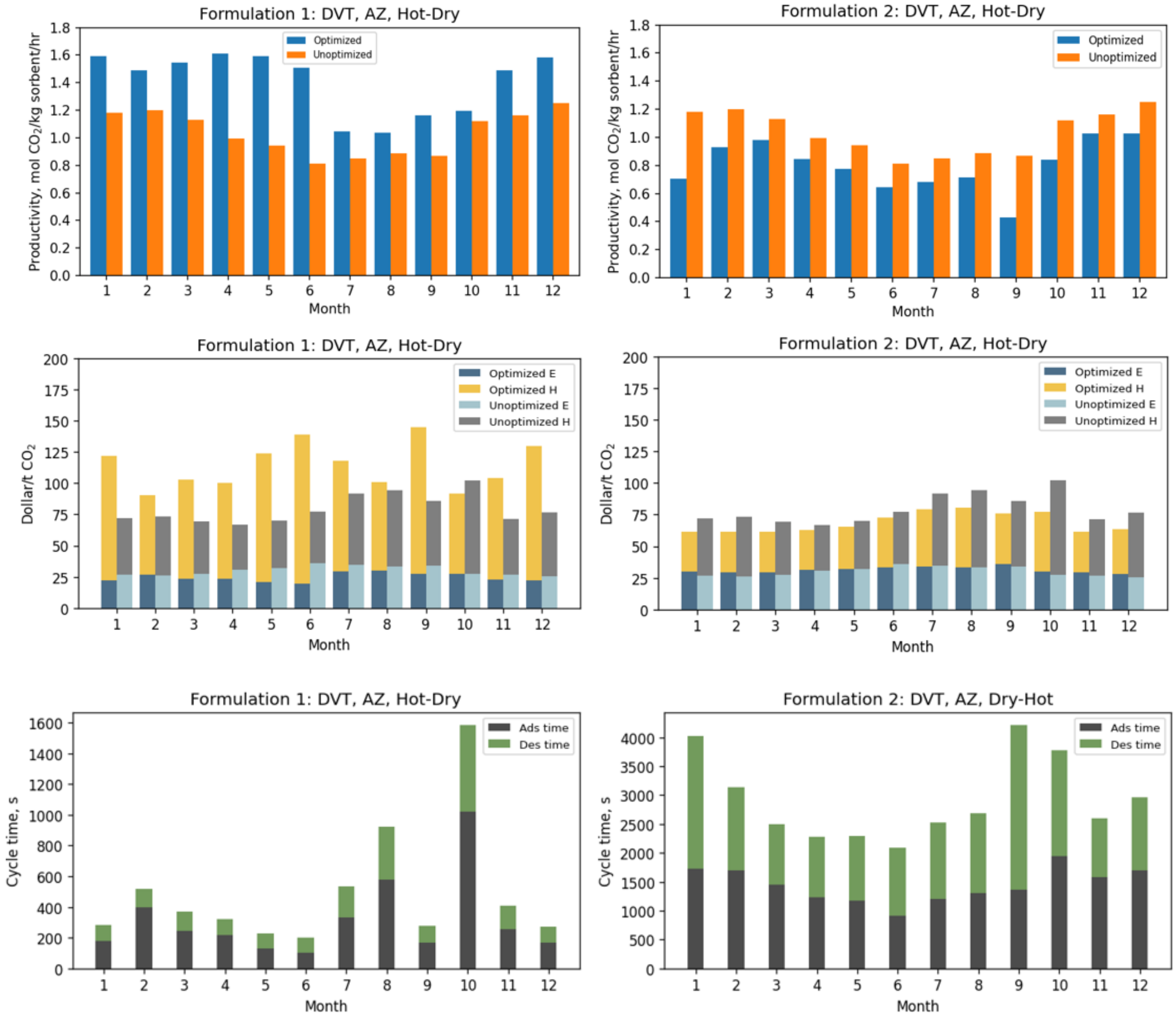


Figure S12. Optimized productivity, energy costs as well as cycle times for DVT, AZ using both optimization formulation.

Table S8. Meteorological inputs and optimized productivity, energy costs as well as cycle times for DVT, AZ using both optimization formulation.

| Meteorological Inputs | | | | | Formulation 1 | | | | | Formulation 2 | | | | |
|---|---|---|---|---|---|---|---|---|---|---|---|---|---|---|
| Month | $CO_2$ (ppm) | $T_{amb}$ (K) | R.H. (%) | $P_{amb}$ (Pa) | Productivity, mol/kg/hr | Electricity, $/tCO2 | Heat $/tCO2 | Adsorption time, s | Desorption time, s | Productivity, mol/kg/hr | Electricity, $/tCO2 | Heat, $/tCO2 | Adsorption time, s | Desorption time, s |
| 1 | 424 | 288 | 31 | 95218 | 1.59 | 22.0 | 99.9 | 177 | 104 | 0.71 | 30.2 | 31.5 | 1729 | 2299 |
| 2 | 423 | 287 | 34 | 94968 | 1.49 | 26.5 | 63.8 | 398 | 122 | 0.93 | 29.1 | 32.5 | 1700 | 1439 |
| 3 | 421 | 291 | 26 | 94836 | 1.54 | 23.3 | 79.6 | 245 | 123 | 0.98 | 29.5 | 32.1 | 1445 | 1045 |
| 4 | 420 | 297 | 15 | 94605 | 1.61 | 23.2 | 77.1 | 219 | 100 | 0.85 | 31.4 | 31.5 | 1224 | 1047 |
| 5 | 418 | 300 | 16 | 94453 | 1.59 | 20.5 | 103.2 | 130 | 100 | 0.77 | 32.2 | 33.4 | 1171 | 1126 |
| 6 | 418 | 305 | 14 | 94364 | 1.51 | 19.7 | 119.3 | 100 | 100 | 0.65 | 33.1 | 39.3 | 918 | 1178 |
| 7 | 415 | 307 | 30 | 94672 | 1.05 | 29.6 | 88.3 | 332 | 204 | 0.68 | 33.6 | 45.6 | 1200 | 1327 |
| 8 | 409 | 305 | 36 | 94618 | 1.03 | 30.0 | 71.1 | 578 | 344 | 0.72 | 33.0 | 47.3 | 1309 | 1376 |
| 9 | 408 | 305 | 27 | 94452 | 1.16 | 27.1 | 117.9 | 168 | 109 | 0.43 | 35.7 | 40.2 | 1359 | 2860 |
| 10 | 414 | 293 | 58 | 94632 | 1.20 | 27.2 | 64.8 | 1023 | 565 | 0.84 | 30.2 | 47.3 | 1949 | 1823 |
| 11 | 425 | 289 | 30 | 94991 | 1.49 | 22.9 | 80.9 | 256 | 152 | 1.03 | 29.5 | 32.3 | 1573 | 1023 |
| 12 | 428 | 285 | 41 | 95055 | 1.58 | 22.0 | 108.1 | 170 | 101 | 1.03 | 28.0 | 35.1 | 1692 | 1267 |

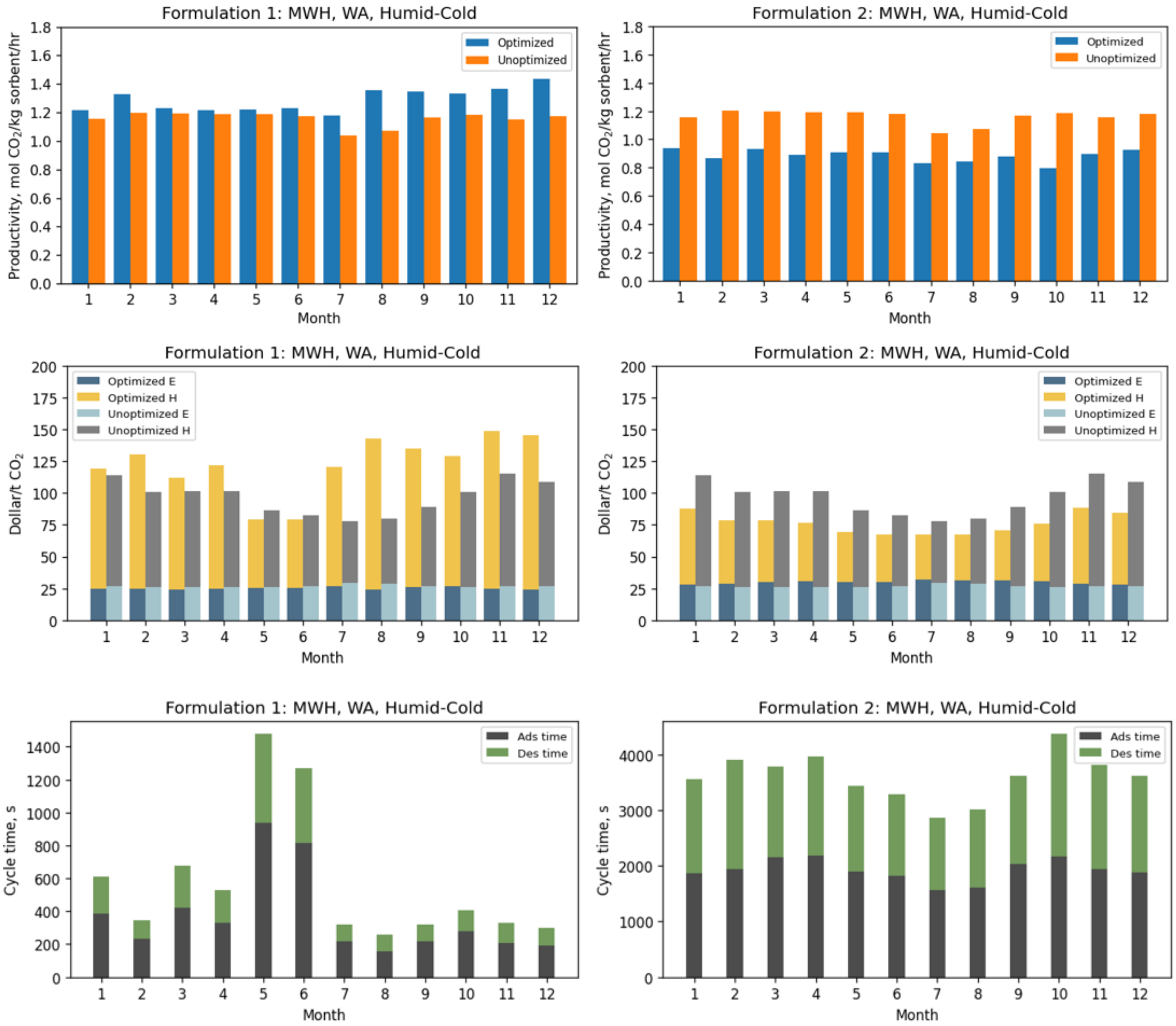


Figure S13. Optimized productivity, energy costs as well as cycle times for MWH, WA using both optimization formulation.

Table S9. Meteorological inputs and optimized productivity, energy costs as well as cycle times for MWH, WA using both optimization formulation.

| Meteorological Inputs | | | | | Formulation 1 | | | | | Formulation 2 | | | | |
|---|---|---|---|---|---|---|---|---|---|---|---|---|---|---|
| Month | $CO_2$ (ppm) | $T_{amb}$ (K) | R.H. (%) | $P_{amb}$ (Pa) | Productivity, mol/kg/hr | Electricity, $/tCO2 | Heat $/tCO2 | Adsorption time, s | Desorption time, s | Productivity, mol/kg/hr | Electricity, $/tCO2 | Heat, $/tCO2 | Adsorption time, s | Desorption time, s |
| 1 | 418 | 274 | 86 | 94199 | 1.22 | 25.0 | 94.4 | 388 | 225 | 0.94 | 28.0 | 60.0 | 1861 | 1694 |
| 2 | 417 | 273 | 77 | 94132 | 1.33 | 24.7 | 105.5 | 233 | 111 | 0.87 | 28.8 | 50.0 | 1950 | 1950 |
| 3 | 417 | 277 | 68 | 93826 | 1.23 | 24.4 | 87.6 | 421 | 258 | 0.93 | 30.2 | 48.4 | 2155 | 1628 |
| 4 | 418 | 281 | 63 | 93920 | 1.22 | 24.5 | 97.3 | 330 | 200 | 0.89 | 30.5 | 46.0 | 2192 | 1769 |
| 5 | 422 | 290 | 48 | 93897 | 1.22 | 25.5 | 53.6 | 935 | 546 | 0.91 | 30.1 | 39.3 | 1900 | 1542 |
| 6 | 416 | 290 | 44 | 93897 | 1.23 | 25.5 | 53.7 | 813 | 456 | 0.91 | 30.2 | 37.4 | 1824 | 1465 |
| 7 | 408 | 296 | 31 | 94145 | 1.18 | 26.4 | 94.5 | 219 | 100 | 0.83 | 32.2 | 35.1 | 1570 | 1298 |
| 8 | 406 | 296 | 35 | 93938 | 1.36 | 24.3 | 118.3 | 158 | 100 | 0.84 | 31.2 | 36.4 | 1608 | 1403 |
| 9 | 405 | 288 | 50 | 93991 | 1.35 | 26.2 | 108.8 | 218 | 102 | 0.88 | 31.1 | 39.5 | 2027 | 1598 |
| 10 | 413 | 282 | 62 | 94259 | 1.34 | 26.5 | 102.4 | 281 | 127 | 0.80 | 30.5 | 45.6 | 2169 | 2213 |
| 11 | 419 | 276 | 81 | 94308 | 1.37 | 24.6 | 124.0 | 208 | 120 | 0.89 | 28.4 | 60.0 | 1941 | 1874 |
| 12 | 420 | 273 | 83 | 94230 | 1.44 | 23.9 | 121.9 | 191 | 108 | 0.93 | 28.2 | 56.2 | 1883 | 1731 |

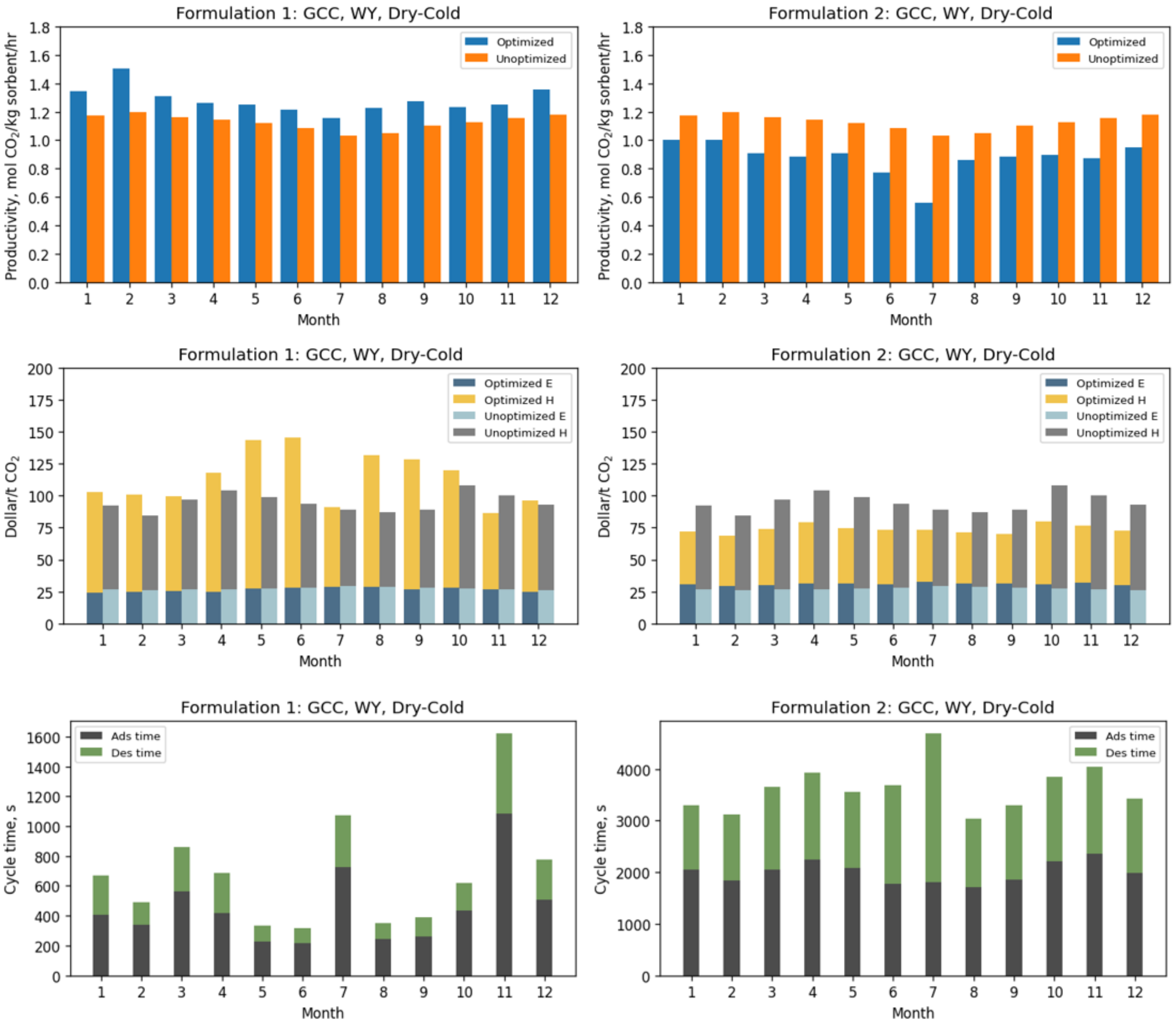


Figure S14. Optimized productivity, energy costs as well as cycle times for GCC, WY using both optimization formulation.

Table S10. Meteorological inputs and optimized productivity, energy costs as well as cycle times for GCC, WY using both optimization formulation.

| Meteorological Inputs | | | | | Formulation 1 | | | | | Formulation 2 | | | | |
|---|---|---|---|---|---|---|---|---|---|---|---|---|---|---|
| Month | $CO_2$ (ppm) | $T_{amb}$ (K) | R.H. (%) | $P_{amb}$ (Pa) | Productivity, mol/kg/hr | Electricity, $/tCO2 | Heat $/tCO2 | Adsorption time, s | Desorption time, s | Productivity, mol/kg/hr | Electricity, $/tCO2 | Heat, $/tCO2 | Adsorption time, s | Desorption time, s |
| 1 | 414 | 270 | 69 | 86906 | 1.34 | 23.8 | 79.1 | 408 | 263 | 1.00 | 30.4 | 42.0 | 2048 | 1245 |
| 2 | 419 | 265 | 70 | 86585 | 1.50 | 24.4 | 76.2 | 342 | 146 | 1.00 | 29.1 | 39.3 | 1844 | 1280 |
| 3 | 418 | 275 | 64 | 86574 | 1.31 | 25.6 | 73.8 | 566 | 296 | 0.91 | 30.1 | 44.0 | 2051 | 1612 |
| 4 | 419 | 278 | 65 | 86647 | 1.26 | 24.9 | 93.3 | 419 | 266 | 0.89 | 31.1 | 47.9 | 2245 | 1683 |
| 5 | 419 | 288 | 55 | 86691 | 1.25 | 27.5 | 116.1 | 229 | 105 | 0.91 | 31.2 | 43.2 | 2091 | 1469 |
| 6 | 418 | 292 | 49 | 86587 | 1.21 | 28.3 | 117.1 | 219 | 100 | 0.77 | 30.4 | 43.1 | 1777 | 1915 |
| 7 | 413 | 297 | 42 | 87133 | 1.16 | 28.9 | 61.9 | 724 | 347 | 0.56 | 32.9 | 40.4 | 1806 | 2895 |
| 8 | 406 | 294 | 41 | 86890 | 1.23 | 28.4 | 103.4 | 243 | 105 | 0.86 | 31.6 | 39.4 | 1707 | 1329 |
| 9 | 407 | 290 | 46 | 86825 | 1.27 | 26.7 | 101.6 | 261 | 129 | 0.88 | 31.0 | 39.3 | 1865 | 1433 |
| 10 | 415 | 280 | 66 | 86949 | 1.23 | 28.3 | 91.8 | 435 | 184 | 0.90 | 30.9 | 49.3 | 2207 | 1645 |
| 11 | 416 | 273 | 70 | 86761 | 1.25 | 26.9 | 59.5 | 1087 | 537 | 0.87 | 32.1 | 44.7 | 2353 | 1688 |
| 12 | 417 | 270 | 69 | 86756 | 1.36 | 24.8 | 71.7 | 506 | 271 | 0.95 | 29.8 | 42.7 | 1981 | 1447 |

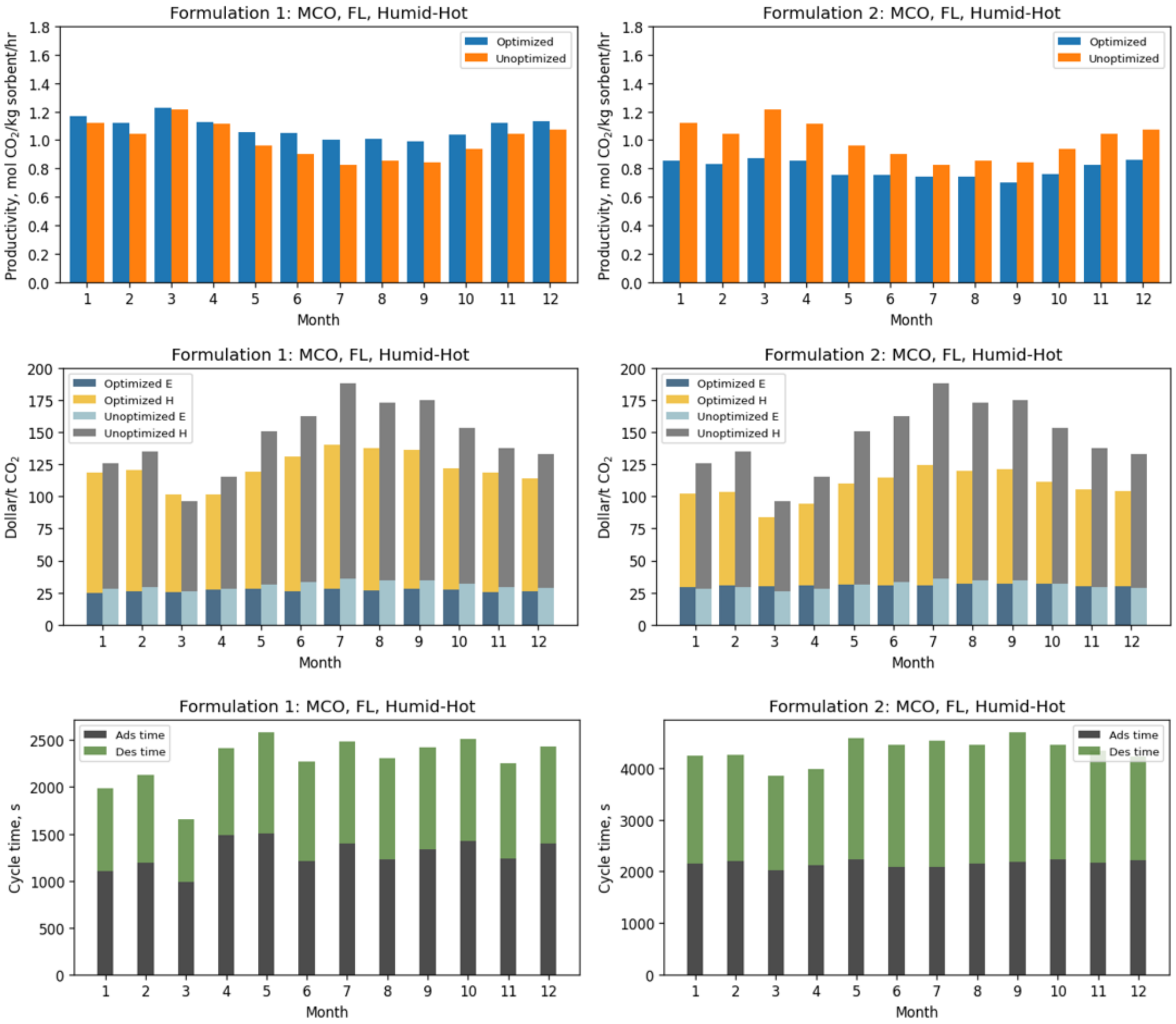


Figure S15. Optimized productivity, energy costs as well as cycle times for MCO, FL using both optimization formulation.

Table S11. Meteorological inputs and optimized productivity, energy costs as well as cycle times for MCO, FL using both optimization formulation.

| **Meteorological Inputs** | | | | | **Formulation 1** | | | | | **Formulation 2** | | | | |
|---|---|---|---|---|---|---|---|---|---|---|---|---|---|---|
| Month | $CO_2$ (ppm) | $T_{amb}$ (K) | R.H. (%) | $P_{amb}$ (Pa) | Productivity, mol/kg/hr | Electricity, $/tCO2 | Heat $/tCO2 | Adsorption time, s | Desorption time, s | Productivity, mol/kg/hr | Electricity, $/tCO2 | Heat, $/tCO2 | Adsorption time, s | Desorption time, s |
| 1 | 419 | 287 | 77 | 102026 | 1.17 | 24.9 | 93.6 | 1106 | 881 | 0.85 | 29.4 | 73.0 | 2149 | 2092 |
| 2 | 424 | 294 | 75 | 102104 | 1.12 | 25.8 | 94.5 | 1194 | 938 | 0.83 | 30.5 | 73.3 | 2196 | 2065 |
| 3 | 424 | 291 | 59 | 101534 | 1.23 | 25.3 | 76.1 | 991 | 668 | 0.87 | 29.8 | 54.1 | 2020 | 1844 |
| 4 | 423 | 295 | 68 | 101427 | 1.13 | 27.5 | 74.2 | 1484 | 934 | 0.85 | 30.5 | 63.6 | 2115 | 1881 |
| 5 | 421 | 297 | 77 | 101423 | 1.05 | 27.8 | 91.7 | 1505 | 1084 | 0.75 | 31.4 | 78.7 | 2227 | 2361 |
| 6 | 439 | 299 | 79 | 101366 | 1.05 | 26.1 | 104.9 | 1212 | 1063 | 0.75 | 30.6 | 84.3 | 2097 | 2355 |
| 7 | 433 | 299 | 83 | 101405 | 1.00 | 28.2 | 112.2 | 1401 | 1084 | 0.74 | 30.5 | 93.8 | 2091 | 2448 |
| 8 | 421 | 299 | 80 | 101570 | 1.01 | 26.9 | 110.5 | 1230 | 1075 | 0.74 | 31.6 | 88.2 | 2149 | 2310 |
| 9 | 414 | 299 | 80 | 101315 | 0.99 | 27.9 | 108.5 | 1337 | 1087 | 0.70 | 32.1 | 88.9 | 2180 | 2530 |
| 10 | 415 | 298 | 77 | 101400 | 1.04 | 27.6 | 94.4 | 1427 | 1086 | 0.76 | 31.9 | 79.3 | 2229 | 2223 |
| 11 | 430 | 293 | 77 | 101517 | 1.12 | 25.4 | 93.5 | 1240 | 1011 | 0.82 | 30.0 | 75.4 | 2171 | 2174 |
| 12 | 425 | 291 | 77 | 101703 | 1.13 | 26.1 | 87.9 | 1400 | 1029 | 0.86 | 30.1 | 74.4 | 2220 | 2020 |

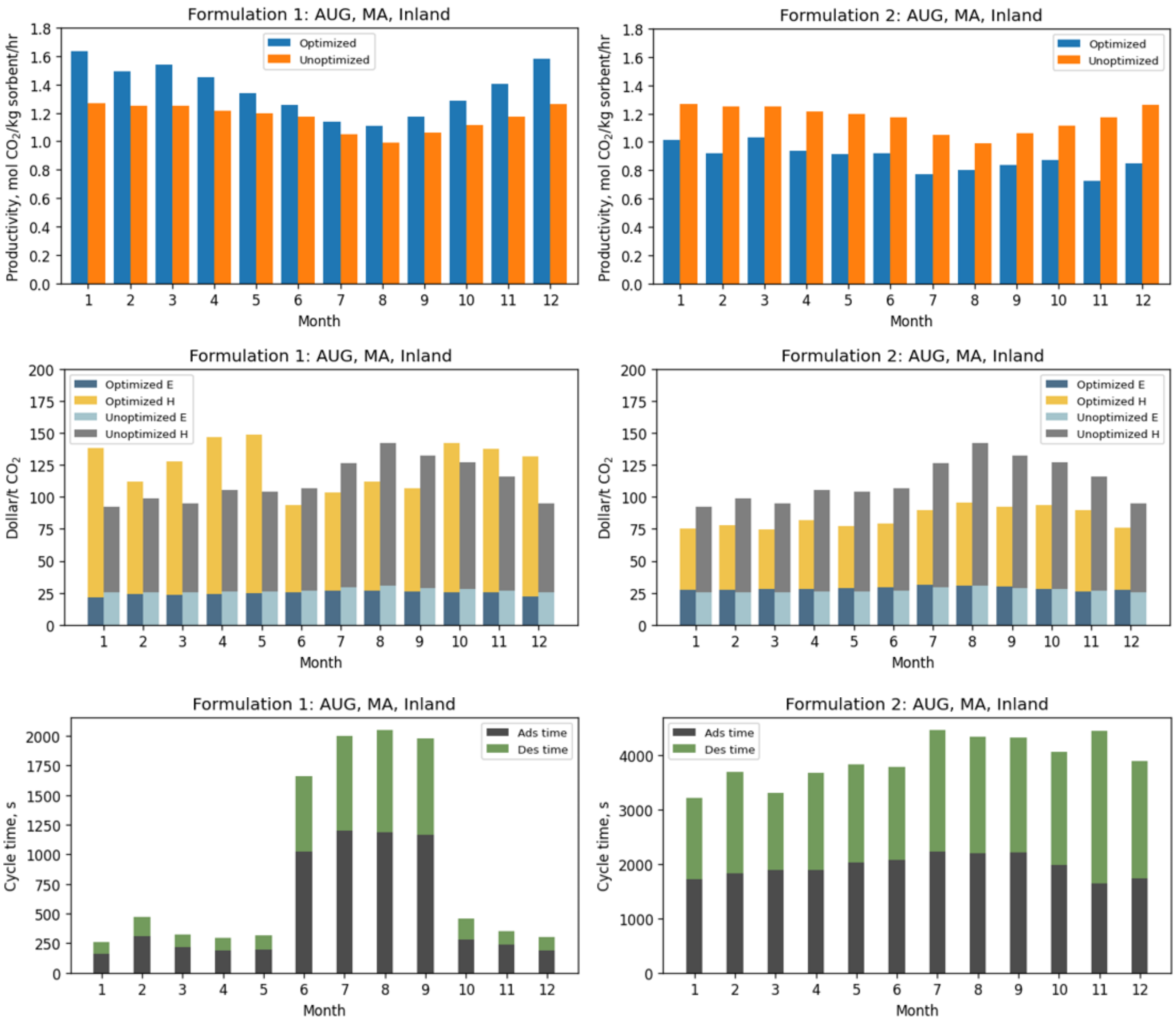


Figure S16. Optimized productivity, energy costs as well as cycle times for AUG, MA using both optimization formulation.

Table S12. Meteorological inputs and optimized productivity, energy costs as well as cycle times for AUG, MA using both optimization formulation.

| Meteorological Inputs | | | | | Formulation 1 | | | | | Formulation 2 | | | | |
|---|---|---|---|---|---|---|---|---|---|---|---|---|---|---|
| Month | $CO_2$ (ppm) | $T_{amb}$ (K) | R.H. (%) | $P_{amb}$ (Pa) | Productivity, mol/kg/hr | Electricity, $/tCO2 | Heat $/tCO2 | Adsorption time, s | Desorption time, s | Productivity, mol/kg/hr | Electricity, $/tCO2 | Heat, $/tCO2 | Adsorption time, s | Desorption time, s |
| 1 | 417 | 267 | 86 | 100118 | 1.63 | 21.5 | 116.9 | 159 | 100 | 1.02 | 27.1 | 47.9 | 1718 | 1496 |
| 2 | 420 | 272 | 79 | 100399 | 1.49 | 23.7 | 88.5 | 309 | 163 | 0.92 | 27.6 | 50.2 | 1834 | 1865 |
| 3 | 417 | 273 | 72 | 99469 | 1.54 | 23.4 | 104.6 | 214 | 110 | 1.03 | 28.2 | 46.7 | 1894 | 1409 |
| 4 | 418 | 278 | 73 | 99749 | 1.45 | 24.2 | 122.4 | 190 | 102 | 0.94 | 27.8 | 54.4 | 1892 | 1786 |
| 5 | 420 | 287 | 65 | 99969 | 1.34 | 24.8 | 124.1 | 196 | 118 | 0.91 | 28.9 | 48.4 | 2025 | 1807 |
| 6 | 421 | 290 | 65 | 99639 | 1.26 | 25.5 | 68.1 | 1026 | 637 | 0.92 | 29.5 | 49.5 | 2070 | 1723 |
| 7 | 405 | 295 | 71 | 100183 | 1.14 | 26.9 | 76.4 | 1201 | 800 | 0.77 | 31.5 | 58.0 | 2229 | 2244 |
| 8 | 406 | 295 | 76 | 99918 | 1.11 | 26.7 | 85.2 | 1189 | 866 | 0.80 | 30.9 | 65.0 | 2192 | 2157 |
| 9 | 410 | 290 | 76 | 100543 | 1.17 | 25.9 | 80.7 | 1169 | 813 | 0.84 | 30.1 | 62.0 | 2214 | 2116 |
| 10 | 413 | 281 | 82 | 99867 | 1.28 | 25.4 | 117.0 | 284 | 173 | 0.87 | 28.2 | 65.4 | 1991 | 2066 |
| 11 | 419 | 274 | 89 | 99847 | 1.40 | 25.5 | 112.2 | 237 | 113 | 0.73 | 26.2 | 63.8 | 1642 | 2810 |
| 12 | 420 | 271 | 80 | 100033 | 1.58 | 22.3 | 109.4 | 189 | 111 | 0.85 | 27.1 | 49.0 | 1743 | 2147 |

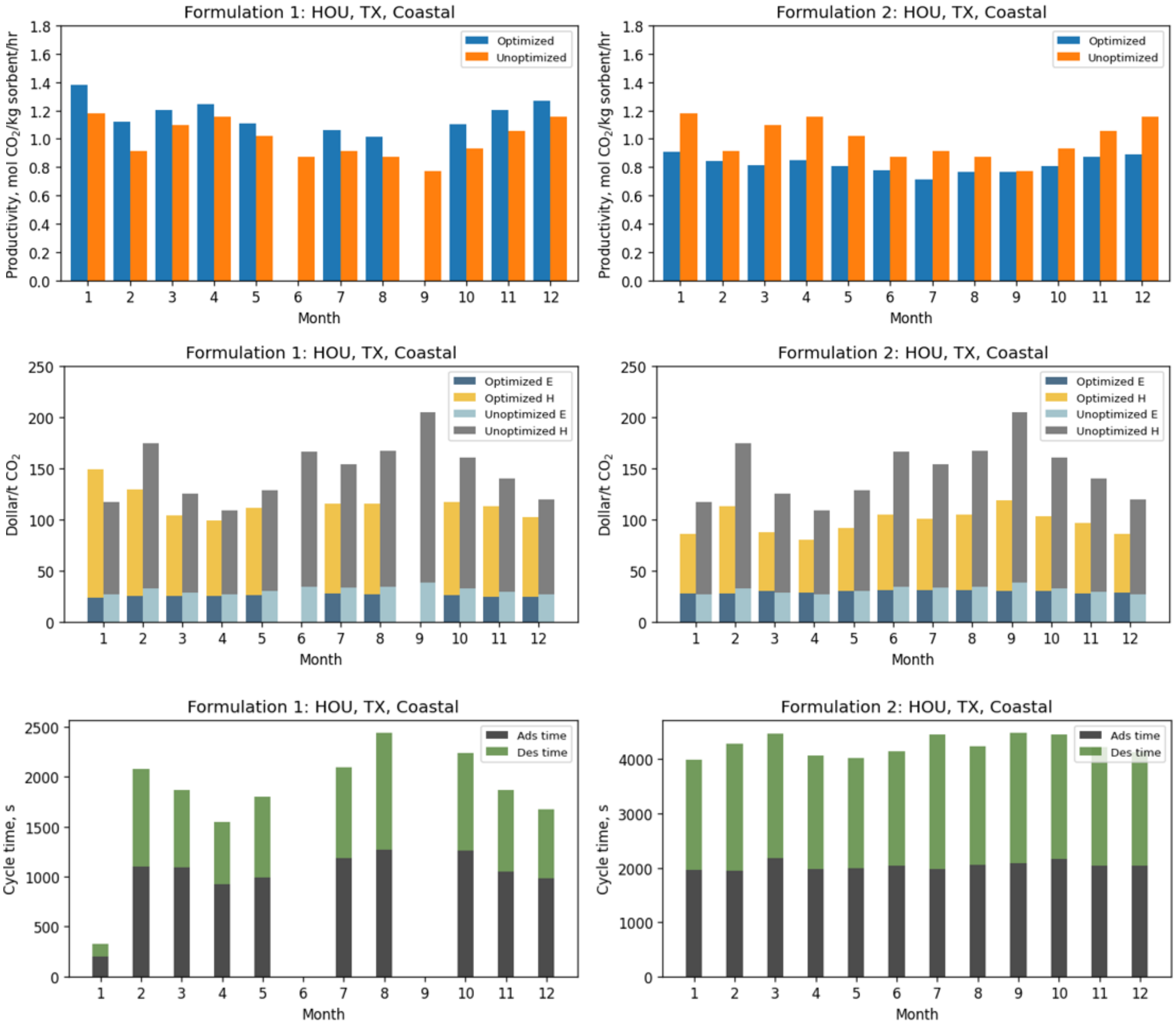


Figure S17. Optimized productivity, energy costs as well as cycle times for HOU, TX using both optimization formulation.

Table S13. Meteorological inputs and optimized productivity, energy costs as well as cycle times for HOU, TX using both optimization formulation.

| **Meteorological Inputs** | | | | | **Formulation 1** | | | | | **Formulation 2** | | | | |
|---|---|---|---|---|---|---|---|---|---|---|---|---|---|---|
| Month | $CO_2$ (ppm) | $T_{amb}$ (K) | R.H. (%) | $P_{amb}$ (Pa) | Productivity, mol/kg/hr | Electricity, $/tCO2 | Heat $/tCO2 | Adsorption time, s | Desorption time, s | Productivity, mol/kg/hr | Electricity, $/tCO2 | Heat, $/tCO2 | Adsorption time, s | Desorption time, s |
| 1 | 428 | 283 | 76 | 102289 | 1.38 | 23.9 | 125.4 | 199 | 128 | 0.91 | 27.6 | 58.3 | 1965 | 2018 |
| 2 | 425 | 291 | 91 | 101654 | 1.12 | 24.8 | 104.5 | 1104 | 972 | 0.85 | 27.3 | 85.9 | 1952 | 2329 |
| 3 | 423 | 292 | 73 | 101415 | 1.20 | 25.3 | 78.4 | 1092 | 772 | 0.81 | 29.7 | 58.2 | 2174 | 2304 |
| 4 | 427 | 293 | 65 | 101360 | 1.24 | 25.2 | 73.8 | 927 | 623 | 0.85 | 28.8 | 51.2 | 1981 | 2091 |
| 5 | 422 | 299 | 70 | 101146 | 1.11 | 25.8 | 85.3 | 995 | 802 | 0.81 | 30.2 | 61.1 | 1989 | 2040 |
| 6 | 420 | 301 | 77 | 101075 | -- | -- | -- | -- | -- | 0.78 | 31.0 | 73.9 | 2043 | 2103 |
| 7 | 428 | 301 | 74 | 101292 | 1.06 | 27.2 | 88.4 | 1187 | 904 | 0.71 | 30.7 | 69.6 | 1982 | 2468 |
| 8 | 421 | 301 | 77 | 101308 | 1.01 | 26.4 | 88.6 | 1273 | 1170 | 0.77 | 31.1 | 73.8 | 2061 | 2179 |
| 9 | 423 | 299 | 86 | 101112 | -- | -- | -- | -- | -- | 0.76 | 29.9 | 88.5 | 2084 | 2409 |
| 10 | 421 | 296 | 81 | 101324 | 1.11 | 26.2 | 91.2 | 1261 | 979 | 0.81 | 29.8 | 73.5 | 2160 | 2288 |
| 11 | 429 | 288 | 82 | 101606 | 1.20 | 24.6 | 88.4 | 1050 | 813 | 0.87 | 27.9 | 68.9 | 2046 | 2184 |
| 12 | 432 | 287 | 74 | 101557 | 1.27 | 24.4 | 78.1 | 981 | 693 | 0.89 | 28.1 | 57.8 | 2045 | 2085 |

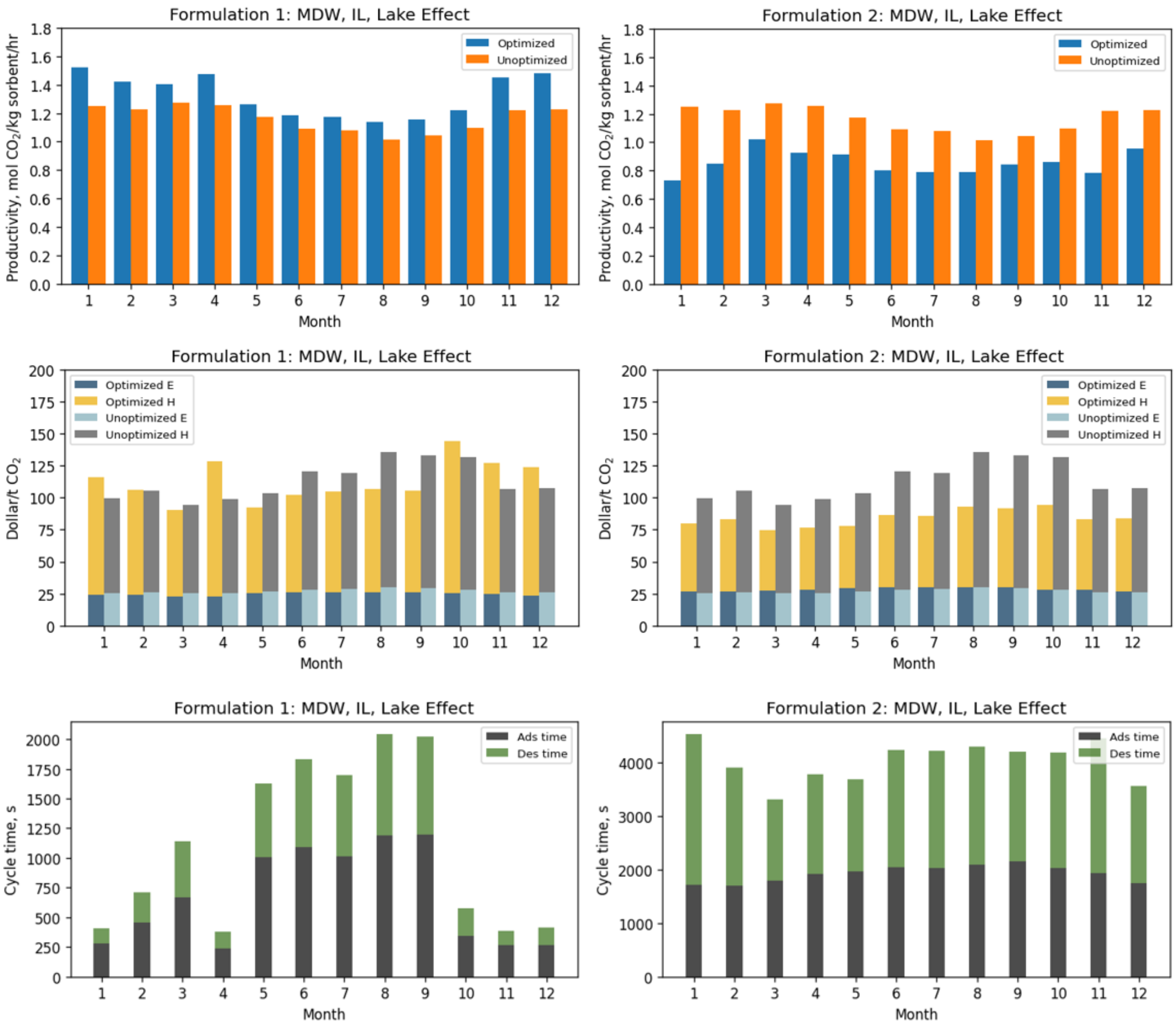


Figure S18. Optimized productivity, energy costs as well as cycle times for MDW, IL using both optimization formulation.

Table S14. Meteorological inputs and optimized productivity, energy costs as well as cycle times for MDW, IL using both optimization formulation.

| **Meteorological Inputs** | | | | | **Formulation 1** | | | | | **Formulation 2** | | | | |
|---|---|---|---|---|---|---|---|---|---|---|---|---|---|---|
| Month | $CO_2$ (ppm) | $T_{amb}$ (K) | R.H. (%) | $P_{amb}$ (Pa) | Productivity, mol/kg/hr | Electricity, $/tCO2 | Heat $/tCO2 | Adsorption time, s | Desorption time, s | Productivity, mol/kg/hr | Electricity, $/tCO2 | Heat, $/tCO2 | Adsorption time, s | Desorption time, s |
| 1 | 426 | 270 | 88 | 99798 | 1.52 | 24.1 | 92.0 | 277 | 131 | 0.73 | 26.7 | 53.1 | 1719 | 2825 |
| 2 | 429 | 272 | 87 | 99703 | 1.42 | 24.2 | 81.6 | 456 | 256 | 0.85 | 26.4 | 56.6 | 1696 | 2220 |
| 3 | 430 | 275 | 68 | 99441 | 1.40 | 22.9 | 67.8 | 670 | 470 | 1.02 | 27.2 | 47.1 | 1797 | 1521 |
| 4 | 432 | 279 | 67 | 99258 | 1.47 | 23.0 | 105.7 | 236 | 146 | 0.92 | 27.9 | 48.9 | 1926 | 1857 |
| 5 | 430 | 293 | 62 | 99041 | 1.26 | 25.6 | 66.7 | 1007 | 619 | 0.91 | 29.2 | 48.7 | 1974 | 1721 |
| 6 | 431 | 296 | 69 | 98928 | 1.18 | 26.0 | 76.3 | 1089 | 740 | 0.80 | 29.8 | 56.7 | 2053 | 2187 |
| 7 | 430 | 297 | 68 | 99322 | 1.17 | 26.4 | 78.4 | 1016 | 680 | 0.79 | 30.0 | 55.9 | 2026 | 2203 |
| 8 | 428 | 297 | 73 | 99146 | 1.14 | 26.3 | 80.5 | 1189 | 858 | 0.79 | 30.3 | 62.6 | 2088 | 2210 |
| 9 | 421 | 294 | 74 | 99474 | 1.16 | 26.2 | 79.5 | 1197 | 825 | 0.84 | 30.1 | 61.5 | 2151 | 2055 |
| 10 | 424 | 285 | 81 | 99342 | 1.22 | 25.6 | 118.4 | 343 | 231 | 0.86 | 28.2 | 66.1 | 2025 | 2162 |
| 11 | 429 | 275 | 80 | 99396 | 1.45 | 25.0 | 101.9 | 263 | 123 | 0.79 | 27.9 | 55.1 | 1930 | 2528 |
| 12 | 439 | 274 | 83 | 99372 | 1.48 | 23.5 | 100.4 | 265 | 149 | 0.95 | 26.5 | 57.2 | 1752 | 1809 |

REFERENCES

(1) Elfving, J.; Sainio, T. Kinetic approach to modelling $CO_2$ adsorption from humid air using amine-functionalized resin: Equilibrium isotherms and column dynamics. *Chemical Engineering Science* **2021**, *246*. DOI: 10.1016/j.ces.2021.116885.

(2) Jacobson, A. R. et al. CarbonTracker, CT2019B. 2020; https://www.esrl.noaa.gov/gmd/ccgg/carbontracker/CT2019B/
